# Genoarchitectonic Compartmentalization of the Embryonic Telencephalon: Insights From the Domestic Cat

**DOI:** 10.3389/fnana.2021.785541

**Published:** 2021-12-16

**Authors:** Nikistratos Siskos, Charalampos Ververidis, George Skavdis, Maria E. Grigoriou

**Affiliations:** ^1^Laboratory of Developmental Biology & Molecular Neurobiology, Department of Molecular Biology & Genetics, Democritus University of Thrace, Alexandroupolis, Greece; ^2^Obstetrics and Surgery Unit, Companion Animal Clinic, School of Veterinary Medicine, Faculty of Health Sciences, Aristotle University of Thessaloniki, Thessaloniki, Greece; ^3^Laboratory of Molecular Regulation & Diagnostic Technology, Department of Molecular Biology & Genetics, Democritus University of Thrace, Alexandroupolis, Greece

**Keywords:** feline brain development, prosomeric model, genoarchitecture, embryonic telencephalon, patterning

## Abstract

The telencephalon develops from the alar plate of the secondary prosencephalon and is subdivided into two distinct divisions, the pallium, which derives solely from prosomere hp1, and the subpallium which derives from both hp1 and hp2 prosomeres. In this first systematic analysis of the feline telencephalon genoarchitecture, we apply the prosomeric model to compare the expression of a battery of genes, including *Tbr1, Tbr2, Pax6, Mash1, Dlx2, Nkx2-1, Lhx6, Lhx7, Lhx2*, and *Emx1*, the orthologs of which alone or in combination, demarcate molecularly distinct territories in other species. We characterize, within the pallium and the subpallium, domains and subdomains topologically equivalent to those previously described in other vertebrate species and we show that the overall genoarchitectural map of the E26/27 feline brain is highly similar to that of the E13.5/E14 mouse. In addition, using the same approach at the earlier (E22/23 and E24/25) or later (E28/29 and E34/35) stages we further analyze neurogenesis, define the timing and duration of several developmental events, and compare our data with those from similar mouse studies; our results point to a complex pattern of heterochronies and show that, compared with the mouse, developmental events in the feline telencephalon span over extended periods suggesting that cats may provide a useful animal model to study brain patterning in ontogenesis and evolution.

## Introduction

Genoarchitectonics, by combining gene expression data with cell morphology and topology, has become in the past decade a powerful approach in the study of the nervous system (Puelles and Ferran, 2012). New ontogenetic construction of the brain of the mouse and other species have been described using genoarchitecture, leading to the revised prosomeric model and the relevant developmental ontology (Medina, 2007; Puelles et al., 2013; Watson et al., 2017). The telencephalon, the largest compartment of the mammalian central nervous system is a highly complex structure in terms of cytoarchitecture, hodology, and function; it derives from the alar plate of the secondary prosencephalon, a domain that corresponds to the anterior territory of the neural plate which, through the differential expression of several regulatory genes is subdivided into two morphological and molecular distinct divisions, namely, the pallium and the subpallium (Puelles et al., 2000; Puelles and Rubenstein, 2003, 2015; Watson et al., 2017). The telencephalon derives from prosomeres hp1 and hp2; the dorsal part of the former produces the evaginated alar plate and gives rise to the entire pallium and most of the subpallium, while the latter gives rise to the non-evaginated alar plate and produces the rest of the subpallium (Watson et al., 2017).

The pallium represents the anlagen of all the cortical areas (e.g., isocortex, allocortex, hippocampus, olfactory bulb) and pallial nuclear masses, such as the claustrum (Cl) and the pallial amygdalar complex, and is molecularly identified by the expression of several genes, for instance, *Emx1/2*, *Pax6*, *Lhx2/9*, *Tbr1/2*, *Nr4a2* (*Nurr1*), and *Lef1*. Within the pallium, four, radially arranged distinct territories have been defined by differential gene expression studies: (1) The ventral pallium (VP), the anlage of the olfactory bulb (OB), and the olfactory allocortex that expresses *Pax6*, *Dbx1*, *Sfrp2*, and *Lhx9* (Puelles et al., 2000; Kim et al., 2001; Medina et al., 2004; García-López et al., 2008). (2) The lateral pallium (LP) is uniquely identified by claustral *Nr4a2* expression and corresponds to the primordium of the claustroinsular complex (Puelles, 2014; Puelles et al., 2016a). (3) The dorsal pallium (DP), the isocortical anlage that expresses, among other genes, *Lhx2*, *Emx1/2*, and *Pax6* (Puelles et al., 2000; Abellán et al., 2014). (4) The medial pallium (MP), the hippocampal primordium is indicatively identified by *Lef1* and *Lhx2/9* expression (Abellán et al., 2014). This tetrapartite model has been successfully applied to study pallial development in a wide range of vertebrates, including various tetrapod species (Medina et al., 2005; Puelles, 2017). Recent evidence in lacertids has proposed the existence of two additional domains, the dorsolateral and ventrocaudal pallia (Desfilis et al., 2018). Integrating these domains into the developmental ontology may upgrade our current understanding of the pallium as being hexapartite (Medina et al., 2019, 2021).

The subpallium, the primordium of the telencephalic basal ganglia contributes to the extended amygdala (EA), the medial, lateral, caudal ganglionic eminences (CGEs), the preoptic area, and the septum (Medina and Abellán, 2012). Moreover, it is the cradle of the cortical GABAergic interneurons (Lavdas et al., 1999). In contrast to the pallium, in the subpallium several genes, for instance, *Gsx1/2*, *Dlx1/2*, *Mash1 (Ascl1)*, *Nkx2-1*, and *Lhx6/7*, are expressed with a unique pattern (Flames et al., 2007). Genoarchitectonics divide the subpallium into four distinct histogenetic domains (Puelles et al., 2013): (1) The striatal (Str) division with major derivatives, the caudoputamen complex and the nucleus accumbens (Acb), molecularly defined by the presence of *Gsx2*, *Dlx2*, and *Mash1*, being devoid of *Nkx2-1* expression. (2) The pallidal (Pd) domain, the anlage of the globus pallidus (GP) is mainly characterized by high *Nkx2-1* along with *Dlx2*, *Mash1*, *Gsx1*, *Lhx6* expression. (3) The preoptic area (POA), the anlage of the postnatal preoptic nuclei, that arises from hp2, corresponds to the non-evaginated telencephalon and uniquely expresses *Shh*. (4) The diagonal domain (Dg), interposed between the POA and the Pd, identified by the expression of *Er81* in the vz which is devoid of *Shh* messenger RNA (mRNA); Dg is also associated with the specification of Somatostatin expressing neurons, and its derivatives include the postnatal nuclei of the diagonal band (DB) and the substantia innominata (Puelles et al., 2016b). Finally, in each histogenetic domain, four secondary subdivisions (i.e., the septal, the paraseptal, the central, and the amygdaloid subdivision) have been recognized along the septoamygdalar axis (Puelles et al., 2013).

The vast majority of the developmental genoarchitectonic studies have been carried out in mice, while data in other mammalian species are very scarce. The domestic cat (*Felis catus*) has been a popular animal model for biological research (Scott, 1977). In neurosciences in the second half of the 20th century, cats have been extensively used to study various aspects of the biology of the central nervous system (CNS), including the physiology of the Cl (for review please refer to Sherk, 2014) or the hodology of the amygdala (e.g., Krettek and Price, 1978a,b), while pioneering atlases were published (Jasper and Ajmone-Marsan, 1954; Markowitsch and Pritzel, 1977). Of particular interest are considered works from the Shatz group that studied neurogenesis in the feline telencephalon (Luskin and Shatz, 1985b), including birth-dating the first neurons generated in the visual cortex (Luskin and Shatz, 1985a). In the 90s, however, the use of cat as a model organism declined (Manger et al., 2008); yet the sequencing of the feline genome revived the interest toward this species (Pontius et al., 2007; Buckley et al., 2020); this may also be attributed to the fact that it can be used as a model organism for several human diseases (e.g., FIV/FAIDS, FeCoV, or SARS-COV-2) and to develop and/or test novel molecular therapeutics (for instance see Bradbury et al., 2013; McCurdy et al., 2015; Gray-Edwards et al., 2017; Shi et al., 2020). In neurosciences, the domestic cat has been recently used in functional neuroimaging studies (Stolzberg et al., 2017), auditory or visual neurosciences (Butler et al., 2015; Kremkow et al., 2016; Galuske et al., 2019), neurophysiology (Darch et al., 2020), as well as neuroanatomy (Hinova-Palova et al., 2019). From a phylogenetic perspective, Laurasiatheria, the superorder from which felids sprouted, diverged before the separation of the human Euarchonta stem progenitor, from the murine Glires ancestor (Bininda-Emonds and Hartmann, 2017). The feline brain, in contrast with the lissencephalic mouse brain, is gyrencephalic (Zilles et al., 2013); this trait favors comparisons both with primates, as well as with the stem mammalian ancestor (Kelava et al., 2013; Catania, 2017). Additionally, felids can be regarded as an organism that provides a “bridge” between rodents and primates (Bradbury et al., 2013; Stolzberg et al., 2017); thus the study of the development of the feline brain not only can provide novel insights into the evolution of the mammalian brain but can also be useful for basic and translational neuroscience (e.g., in Graff et al., 2020).

In this work we have studied the genoarchitectonic organization of the embryonic feline telencephalon by analyzing the expression of several gene markers, the orthologs of which have been previously used in other species, to identify specific domains (e.g., Puelles et al., 2000; Flames et al., 2007; Abellán et al., 2014; Desfilis et al., 2018). We characterized, within the pallium and the subpallium, domains, and subdomains topologically equivalent to those that have been previously described in other vertebrate species and we studied the timing and duration of several developmental events. Our data show that the genoarchitectural map of the feline telencephalon is highly conserved. When compared with data from mouse studies heterochronies in the timing of developmental events were observed; moreover, developmental processes in the feline telencephalon span over extended periods suggesting that cats may provide a useful animal model for increasing our knowledge on the degree of conservation and divergence in brain patterning and morphogenesis throughout ontogenesis and evolution.

## Materials and Methods

### Animals

Feline (*Felis catus*) embryos or fetuses were obtained from domestic cats referred to the Unit of Obstetrics and Surgery of the Companion Animal Clinic of the School of Veterinary Medicine, Faculty of Health Sciences, Aristotle University of Thessaloniki. More specifically, clinically healthy female cats admitted to the Unit for preventive ovariohysterectomy were screened; in case of pregnancy, the excised gravid uterus was not processed for incineration, but immediately incised. The anesthetic protocol included: premedication with combined dexmedetomidine (25 μg kg^–1^ im) and butorphanol (0.1 mg kg^–1^ im), analgesia with meloxicam (0.1 mg kg^–1^ sc), anesthesia induction with ketamine (10 mg kg^–1^ im), and maintenance with ketamine (4-6 mg kg^–1^ iv). Standard operational procedures were followed for midline approach ovariohysterectomy.

Embryos (or fetuses) were collected, rinsed in cold Phosphate Buffer Saline (PBS), and were either immediately frozen (−80°C; tissues intended for RNA/DNA extraction), or fixed with 4% w/v paraformaldehyde (PFA) in PBS for (at least) 24 h at 4°C. Following fixation, tissues were washed with PBS, cryoprotected in 30% w/v sucrose in PBS, embedded in the appropriate sectioning plane (coronal, horizontal, or sagittal) using Tissue Freezing Medium (Leica, Germany), and stored at −80°C until sectioning. Sections (12 μM) were generated using a Leica CM1900UV cryostat, collected on Superfrost plus (Fisher Scientific, United States) slides, air-dried for at least 30 min, and stored at −80°C until later use.

Gestational age was initially assessed during the pre-surgical physical examination, as well as by examining macroscopically the gravid uterus. Given this first approximate estimation, embryos and fetuses were staged after fixation, according to their morphology and crown-rump length (Evans and Sack, 1973; Knospe, 2002). As embryos and fetuses were not collected from timed pregnancies, crown-rump length measurements and morphology do not allow for the accurate evaluation of the gestational age. Thus, we grouped embryos and fetuses in classes spanning two consecutive days, as follows: E22/23 (*n* = 7), E24/25 (*n* = 12), E26/27 (*n* = 21), E28/29 (*n* = 3) and E34/35 (*n* = 5).

### Riboprobes

The DNA fragments used for the generation of riboprobes were amplified either from genomic DNA (gDNA) or from E26/27 feline brain complementary DNA (cDNA). Primers (Supplementary Table 1) were designed using the National Center for Biotechnology Information (NCBI) Primer Blast tool^1^, based on the feline genomic/cDNA sequences published in GenBank. Amplicons were cloned (following suitable digest to create “sticky” or “blunt” ends, or further minor modifications; for details, please refer to the Supplementary Table 2) in pBluescript II KS(+) (Agilent/Stratagene, United States). All constructs were verified by sequencing (Starseq, Germany). Linearized plasmids were used for *in vitro* transcription of antisense RNA probes with T3 or T7 RNA polymerase (Takara, Japan), according to manufacturer’s instructions, using Digoxigenin-11-UTP (Roche, Switzerland). For *Nkx2-1*, we used the murine probe (Shimamura et al., 1995), given that the identity between the murine and feline homologs is high (96%).

### *In situ* Hybridization

*In situ* hybridization was performed as previously described (Stylianopoulou et al., 2016). Slides were mounted in Glycergel (DAKO) and photographed with a Leica DM5500 B (Leica Microsystems) microscope equipped with a DFC7000T or a DFC310FX digital camera (Leica Microsystems). Images were captured using the camera software (LAS v4.13, Leica Microsystems); image panels and schemata were created with the GIMP (gimp.org).

## Results

### Timing of Patterning and Cell Specification in the Feline Telencephalon

In murine embryos, patterning and cell specification events mainly occur between E11 and E14.5 (for review please refer to Guillemot, 2005; Martynoga et al., 2012), while most major genoarchitectonic studies (e.g., Puelles et al., 2000, 2016a; Medina et al., 2004; Flames et al., 2007; García-López et al., 2008; Abellán et al., 2014) have been performed in E12.5-13.5 embryos. As already mentioned in the materials and methods, the feline embryos and fetuses used in this work were obtained from domestic cats and the gestational age was assessed according to their morphology and crown-rump length (Evans and Sack, 1973; Knospe, 2002). To define the developmental stages that would correspond to the murine E12.5-13.5; we used the prediction model of neural development developed by Workman et al. (2013). Key events (Supplementary Table 3) of the development of the cortex, the limbic system, and the striatum were used to translate developmental time between mouse and cat embryos. The model predictions indicated a rough equivalence of the murine stages E11, E12, and E13/14 to feline E22/23, E24/25, and E26/27, respectively, these results were further corroborated by studying the general morphology of the feline brain on sections. Given that two earlier works (Luskin and Shatz, 1985a,b) on feline embryos and fetuses had demonstrated that the first neurons within the pallium are generated between E24 and E30, we chose to focus primarily on the E26/27 feline embryonic telencephalon. Embryos of earlier (E24/25 and E22/23) or fetuses of more advanced (E28/29, E34/35) developmental stages were occasionally used for corroboration purposes and to temporally map developmental events.

We then utilized *in situ* hybridization with markers for various telencephalic domains that have been established in other species (mainly in the mouse) namely: *Lhx2*, *Lef1* (Abellán et al., 2014), *Emx1*, *Tbr1*, *Pax6* (Puelles et al., 2000), *Nr4a2* (*Nurr1*; Puelles et al., 2016a) *Tbr2* (*Eomes*; Englund et al., 2005), *Dlx2*, *Nkx2-1*, *Lhx6*, *Lhx7*, *Er81* (Sussel et al., 1999; Flames et al., 2007; García-López et al., 2008), *Gad2* (Katarova et al., 2000), and *Mash1* (Casarosa et al., 1999). To this end appropriate gene segments (Supplementary Table 2), were amplified by PCR (Supplementary Table 1) from feline gDNA or cDNA (transcribed out of E26/27 head total RNA) and cloned into suitably prepared vectors. Recombinant plasmids were used to generate DIG-labeled riboprobes through *in vitro* transcription. The analysis of the results was performed using published atlases (Jacobowitz and Abbott, 1997; Schambra, 2008; Paxinos and Ashwell, 2018) and articles (Medina et al., 2004; Flames et al., 2007; García-López et al., 2008; Abellán et al., 2014; Puelles et al., 2016a), as well as online resources (Allen Brain Atlas^2^; GenePaint)^3^ on the developing mouse or rat brain.

### The Pallium and the Subpallium

To delineate the proliferative zones of the pallium and the subpallium, along the rostrocaudal axis, we compared the expression patterns of *Tbr2* and *Dlx2* in E26/27 embryos. In the mouse, the pallial proliferative zones strongly express *Tbr2* (Bulfone et al., 1999), a characteristic attributed to the population of basal intermediate progenitors of glutamatergic neurons that constitute the pallial svz (Englund et al., 2005); *Dlx2* is strongly expressed by the populations of the (non-glutamatergic) progenitors of the subpallium, demarcating, thus, the extent of the subpallial svz/vz (Eisenstat et al., 1999). In several studies *Pax6* or *Tbr1* have been used to demarcate the pallium and *Gsx2* or *Dlx2* the subpallium (Puelles et al., 2000; Yun et al., 2001; Stenman et al., 2003b; Carney et al., 2009; Desfilis et al., 2018); we chose, however, *Tbr2* over *Pax6* as the latter expands into the subpallial striatal division (Yun et al., 2001; Flames et al., 2007), hampering the delineation of the pallial domain. Moreover, we chose *Dlx2* over *Gsx2*, as the former is expressed by cells residing in both proliferative zones (Eisenstat et al., 1999). *Tbr1*, on the other hand, is a well-known marker of pallial post-mitotic neurons (Bulfone et al., 1995; Englund et al., 2005), we, therefore, considered that we cannot use it as we could not perform a direct comparison of its expression with the *Dlx2* expression pattern.

**FIGURE 1:**
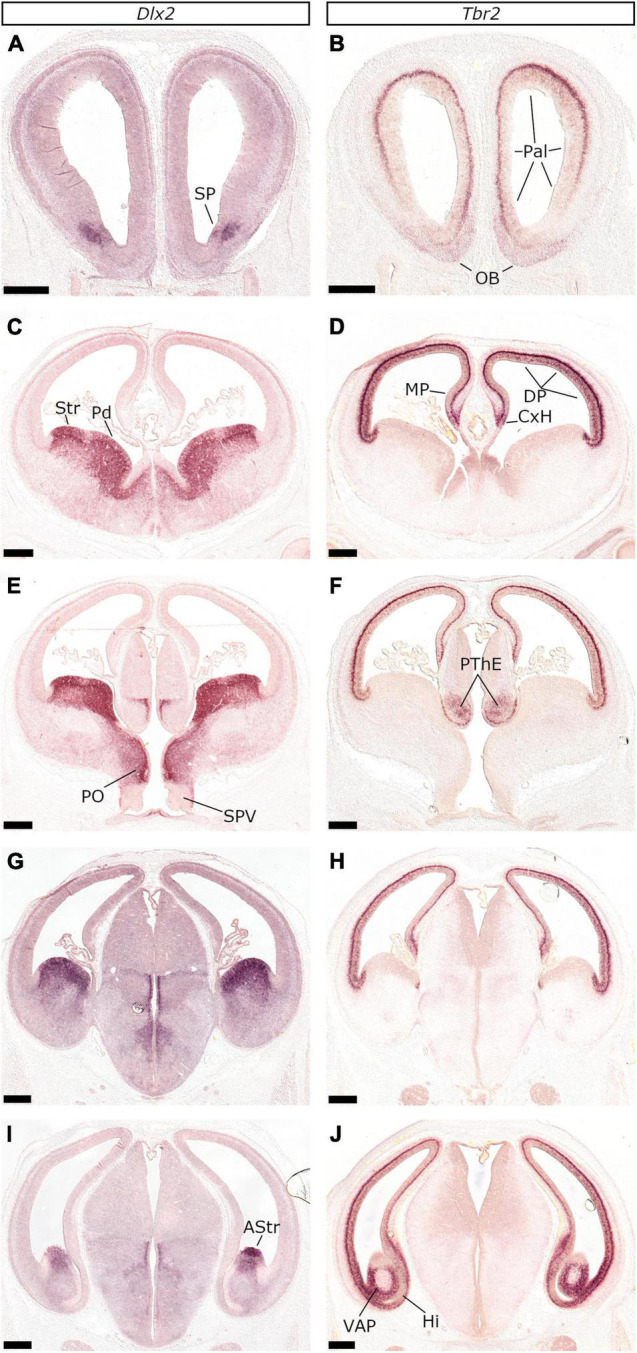
*Tbr2* and *Dlx2* expression demarcate the proliferative zones of the feline pallium and subpallium along the rostrocaudal axis. *In situ* hybridization on coronal sections of E26/27 embryos with *Tbr2* **(A,C,E,G,I)** or *Dlx2* **(B,D,F,H,J)** probes. Scale bars 500 μm.

**FIGURE 2:**
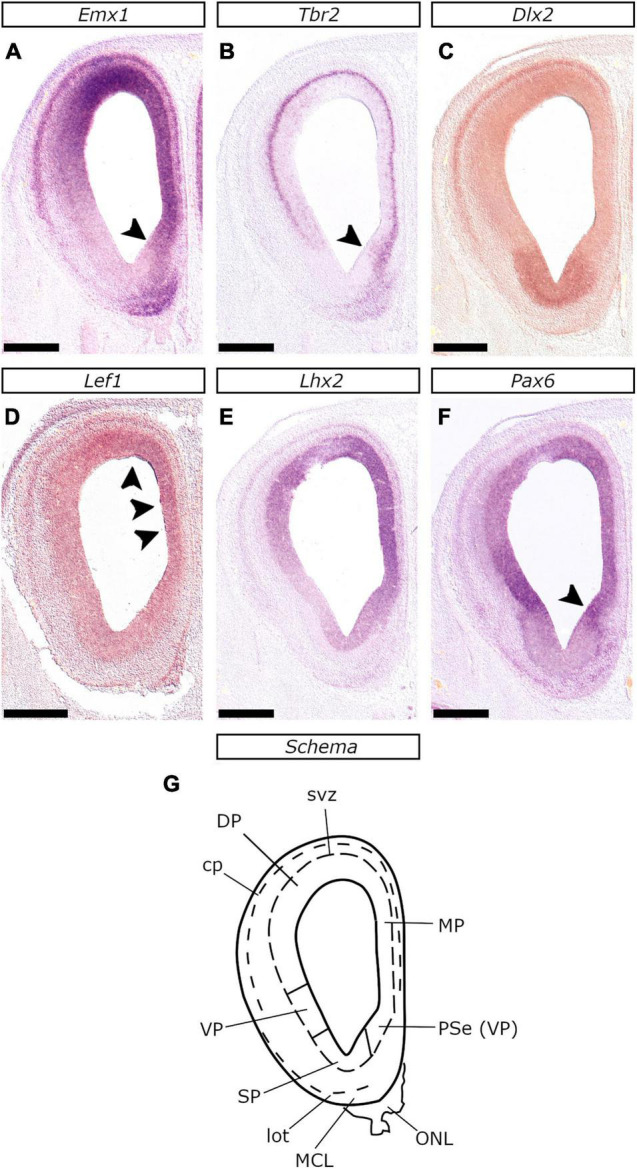
Organization of the E26/27 telencephalon at the retrobulbar level. *In situ* hybridization on coronal sections with *Emx1* **(A)**, *Tbr2* **(B)**, *Dlx2* **(C)**, *Lef1* **(D)**, *Lhx2* **(E)**, and *Pax6* **(F)** probes. **(G)** Schematic representation of the plane of section corresponding to the coronal sections shown in **(A–F)**. The ventricular zone (vz) of the ventral pallium (VP) expressed high levels of *Pax6*, low levels of *Lhx2*, but lacked *Emx1* expression (compare **F**, **E**, and **A**). Arrowheads in **(A,B,F)** indicate the ventral region of the pallial septum (PSe) that expressed high levels of *Pax6* **(F)** and *Tbr2* **(B)**, along with low levels of *Emx1*; this region was considered to be part of the VP. Arrowheads in **(D)** indicate *Lef1* expression in the vz of the dorsal and the medial (dorsal-most region) telencephalic wall. Note the expression of *Emx1* and *Tbr2* within the mitral cell layer (MCL). Scale bars 500 μm.

**FIGURE 3:**
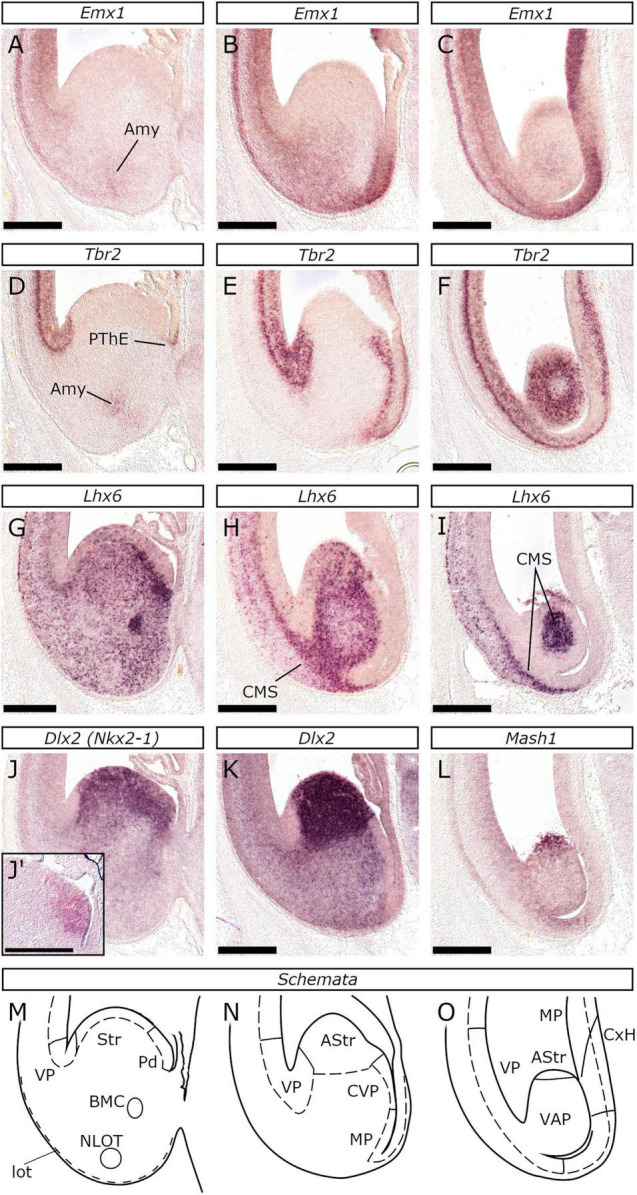
At posterior levels, VP expanded at the expense of subpallium which became gradually restricted. *In situ* hybridization at three successive coronal levels of E26/27 embryonic brain posterior to the internal capsule. At the first level shown in **(A,D,G,J,J′)** and in the schematic representation **(M)**, the VP could be identified in the lateral wall by *Tbr2* expression in the svz and the vz **(D)** and the absence of *Emx1* in the vz **(A)**. The subpallium consisted at this level of both Str and Pd domains, as indicated by *Nkx2-1* expression in the vz/svz **(J′)** and *Lhx6* in the svz **(G)** within the medial aspect of the *Dlx2* labeled **(J)** domain. *Tbr2* expression was also observed in the prethalamic eminence (PThE), while both *Tbr2* and *Emx1* expression marked the amygdala primordium (Amy in **A** and **D**). *Lhx6* expression further demarcated the basal magnocellular complex (BMC) while the nucleus of the lateral olfactory tract (NLOT) primordium appeared as a superficial *Lhx6*-negative focus **(G,M)**. At the second level shown in **(B,E,H,K)** and in the schematic representation **(N)** the pallium extended in the medial aspect of the ventral telencephalon and the subpallium restricted. *Tbr2* expression was observed in both proliferative zones of this pallial domain **(E)**; *Emx1* expression appeared to divide it into a dorsal *Emx1-*negative and a ventral *Emx1*-expressing subdomain **(B)**. The dorsal area corresponds to the central VP (CVP), while the ventral represents the MP **(N)**. At this level, *Nkx2-1* was not expressed (data not shown), thus the caudal subpallium is striatal in nature corresponding to the AStr **(N)**. *Lhx6*-expressing neurons **(H)** exit the ventral telencephalon and migrate through the pallial svz/iz; this represents the caudal migratory stream (CMS). At the third level shown in **(C,F,I,L)** and in the schematic representation **(O)** the entire ventral telencephalon expressed *Tbr2* **(F)** but not *Emx1* **(C)** and it corresponded to the vz of the VAP. The subpallium (AStr) was restricted to the dorsal-most tip, as revealed by *Mash1* expression **(L)**. The core of the VAP showed high *Lhx6* expression **(I)**, which is attributed to the migrating interneurons of the CMS. Scale bars 500 μm.

Rostrally, around the coronal level where the fila olfactoria are visible, almost the whole telencephalon expressed *Tbr2*, apart from a lateroventral, spot-like, *Tbr2*-negative, region that showed high *Dlx2*-expression (Figures 1A,B); this suggested that rostrally, the telencephalon was genoarchitectonically pallial, except for a well-demarcated focus in the lateral-ventral wall corresponding to the rostral-most end of the striatal partition. More posterior planes revealed that the *Dlx2*-expressing territory expanded ventrally toward the medial telencephalic wall (the septal division of the striatal partition; compare Figures 1A,B, 2B,C and Supplementary Figures 1A, B) at the expense of the *Tbr2* expression domain. Furthermore, *Dlx2* was highly expressed in the proliferative zones of the central and amygdaloid divisions of the subpallial partitions (ganglionic eminences) while *Tbr2* was confined dorsally, demarcating the pallial anlagen (Figures 1C–H). Noticeably, the caudal-most end of the ventral telencephalon expressed high levels of *Tbr2* and presented *Dlx2*-expression only in its dorsal-most tip (Figures 1I,J, for close-ups, see Figures 3E,F,K,L). This domain corresponded to the ventropallial amygdalopiriform area (VAP), while the *Dlx2* (Figures 1I, 3K) expressing area was considered to correspond to the vz of the striatal amygdala (AStr).

**FIGURE 4:**
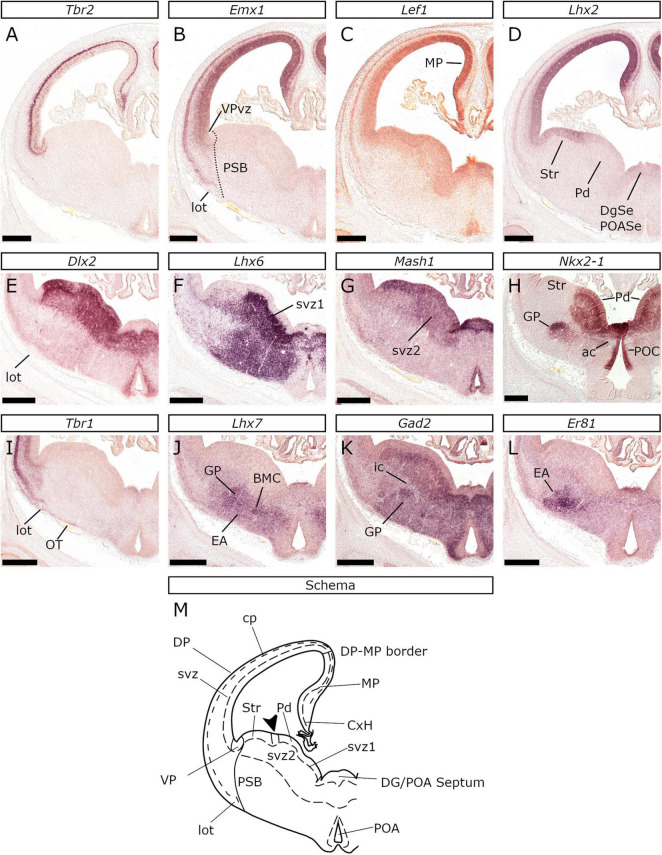
Pallial and subpallial domains of the feline telencephalon at E26/27, at the interventricular foramina (ivf) level. *In situ* hybridization on coronal sections with *Tbr2* **(A)**, *Emx1* **(B)**, *Lef1* **(C)**, *Lhx2* **(D)**, *Dlx2* **(E)**, *Lhx6* **(F)**, *Mash1* **(G)**, *Nkx2-1* **(H)**, *Tbr1* **(I)**, *Lhx7* **(J)**, *Gad2* **(K),** and *Er81* **(L)** probes. **(M)** Schematic representation of the plane of section corresponding to the coronal sections shown in **(A–L)**. The pallium was characterized by *Tbr2, Emx1, Lef1, Lhx2*, and *Tbr1* **(A–D,I)** expression and the subpallium by *Dlx2*, *Lhx6*, *Mash1*, *Nkx2-1*, *Lhx7*, *Gad2*, and *Er81* **(E–H,J–L)**. *Tbr2* **(A)** and *Lhx2* **(D)** but not *Emx1* **(B)** were expressed in the vz of the VP. The medial pallium (MP) was characterized by *Lef1* **(C)**, *Emx1* **(B),** and *Lhx2* **(D)** expression. *Emx1* and *Tbr1* were highly expressed in the cp **(B,I)**. The proliferative zones of the subpallium expressed *Dlx2* **(E)** and *Mash1* **(G)** and were divided into the striatal domain of the subpallium (Str), pallidal domain (Pd), diagonal domain of the subpallium Dg, and preoptic Area (POA) compartments. The vz and the sub-vz (svz) of the Str anlage did not express *Nkx2-1*, in contrast with the Pd, Dg, and POA primordia **(H)**. Note that the vz of the POA and the Dg lacked *Dlx2* expression **(E)**, but expressed *Nkx2-1* **(H)**, *Mash1* **(G),** and weakly *Lhx2* **(D)**. *Lhx6* **(F)**, *Lhx7* **(J)**, *Gad2* **(K),** and *Er81* **(L)** expression was observed in the subpallial mantle as well as focal *Nkx2-1* expression within the GP. The arrowhead in the schema points to the *Dlx2*-negative domain shared between pLGE4 and pMGE1. Scale bars 500 μm.

The mutually exclusive expression of *Tbr2* and *Dlx2* along with *Tbr1* and *Gad2*, assisted us in locating the pallial-subpallial boundary (PSB), in the following locations: (1) In the lateral telencephalic wall, all along the rostrocaudal axis (Schemata in Figures 2–4); (2) In the medial telencephalic wall (septal PSB), caudal to the retrobulbar area, but rostral to the plane of the interventricular foramina (ivf) (Figures 2B,C and Supplementary Figures 1A,B); (3) In the medial aspect of the basal telencephalon, caudal to the positive prethalamic eminence (PThE), between the amygdaloid subpallial division and the VP (or CVP according to Ruiz-Reig et al., 2018; Figures 3D,J,M,E,K,N); (4) In the caudal-most aspect, between the VAP and the AStr (Figures 3F,L,O). As in mice and chicken (Puelles et al., 2000), the PSB could be imagined as a plane connecting the ventricular and pial surfaces, spanning from the *Tbr2*/*Dlx2* abutting vz/svz region to the *Tbr1*/*Gad2* neighboring territory of the lot.

### The Tetrapartite Nature of the Feline Pallium

To delineate molecularly distinct partitions within the feline pallium we studied the combinatorial expression of *Tbr2*, *Emx1*, *Pax6*, *Lhx2*, *Lef1*, *Tbr1*, and *Nr4a2*. Our analysis was based on the tetrapartite model described by Puelles et al. (2000, 2016a).

#### Ventral Pallium

**FIGURE 5:**
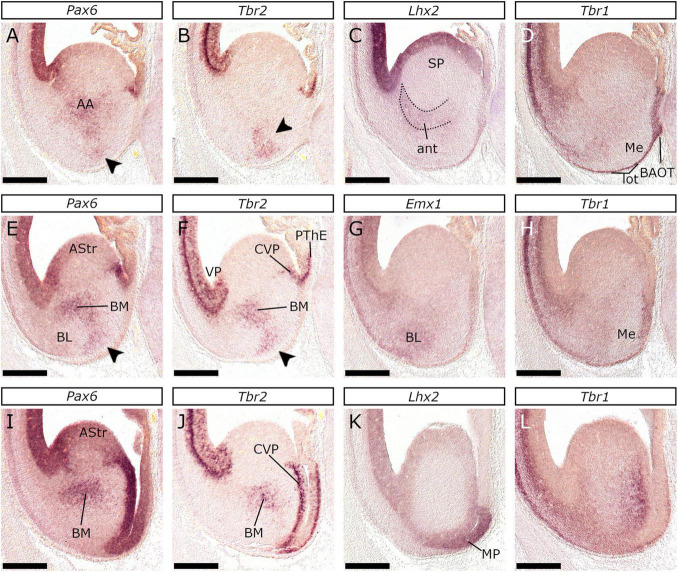
Nuclear organization of the amygdala at E26/27. *In situ* hybridization in three successive coronal planes of the E26/27 embryonic brain with *Pax6* **(A,E,I)**, *Tbr2* **(B,F,J)**, *Lhx2* **(C,K)**, *Tbr1* **(D,H,L)** or *Emx1* **(G)** probes. The first level presented **(A–D)** refers to coronal sections immediately caudal to the ic. The presumed anterior amygdalar radial unit (dotted area in C) expressed *Lhx2*. *Pax6* was expressed in the AA primordium **(A)**, while more diffuse *Pax6* labeling was also observed superficially (arrowhead in **A**), in an area that was further characterized by *Tbr2* expression (arrowhead in **B**). This area is in close association with the lot, that expressed *Tbr1* along with the BAOT primordium **(D)**. Notably, the Me anlage lacked *Tbr1* expression **(D)**. Caudal to this level, *Pax6* **(E)** and *Tbr2* **(F)** were expressed in the BM primordium and the superficial domain (arrowheads) as noted in **(A,B)**. Notably, the *Pax6/Tbr2* expressing area embraced the *Emx1*-expressing BL primordium **(G)**. The superficial domain is thought to represent the ACo primordium, or subpopulations of the *Tbr1*-negative **(H)** Me primordium. At more caudal planes the *Pax6*/*Tbr2*-expressing focus represents the BM anlage **(I,J)**. Note that the pallial vz ventral to the CVP expressed *Lhx2* **(K)** corresponding to the MP. Scale bars 500 μm.

**FIGURE 6:**
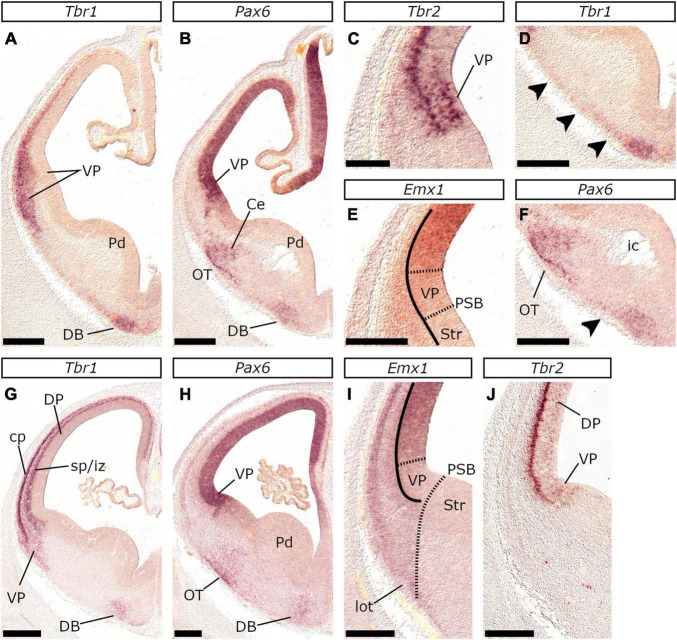
*Tbr1* in the VP mantle was expressed at E22/23 but the lamination of the cp and the sp/iz appeared at E24/25. *In situ* hybridization on coronal sections at E22/23 **(A–F)** and E24/25 **(G–J)** with *Tbr1* **(A,D,G)**, *Pax6* **(B,H)**, *Tbr2* **(C,J)** and *Emx1* **(E,I)** probes. At E22/23 *Tbr1* appeared first in the VP mantle with a VP-high to DP-low gradient **(A)**. *Pax6* **(B)** and *Tbr2* **(C)** were expressed in the vz of the VP, in contrast with *Emx1* **(E)**. *Tbr1* was also present in the diagonal band (DB) anlage in the subpallium **(A,D)**, along with *Pax6* **(B,F)**. Arrowheads in **(D,F)** indicate a migrating, septal-derived, cell population, that expressed low levels of *Tbr1* and *Pax6* and extended subpially between the DB and the VP. *Pax6* expression was detected further in OT **(B,F)**. At E24/25, *Tbr1* **(G)** expression marked the cp and the sp/iz, revealing the lamination of the pallial mantle in contrast with E22/23 (compare **A** with **G**). Lamination was more obvious at the VP-LP mantle; cp and sp/iz converge at the MP and the dorsal-most regions of the DP to a single *Tbr1*-expressing band. At E24/25 the vz of the VP was still *Tbr2* positive **(J)** but lacked *Emx1* expression **(I)**. Notably, *Tbr2* expression was extended beyond the VP, into the vz of the DP (compare **C** and **J**). Scale bars 500 μm, except for **(C)** and **(E)**: 250 μm.

**FIGURE 7:**
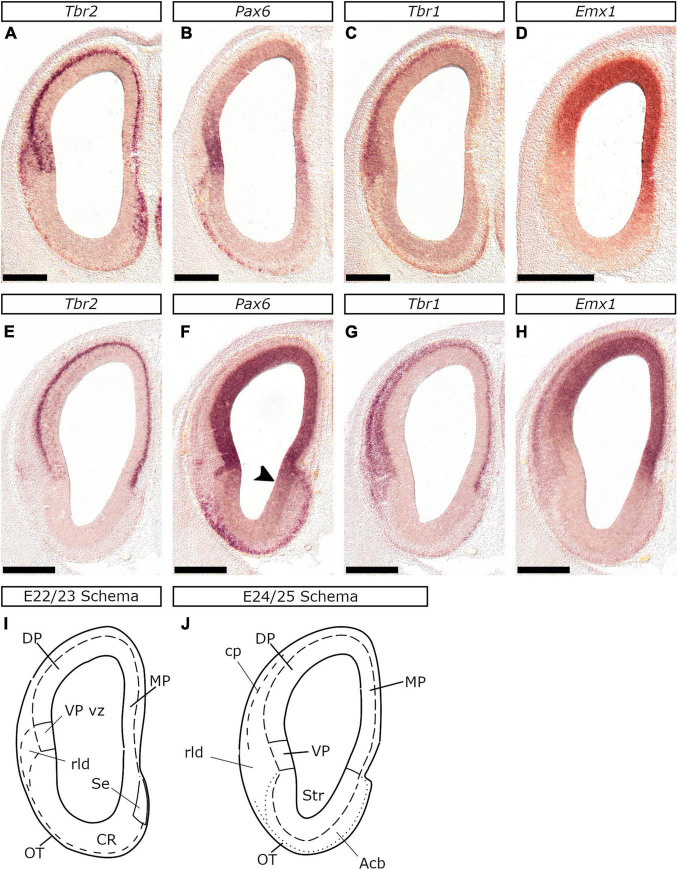
Cajal-Retzius neurons in the retrobulbar telencephalic fields at E22/23 and E24/25. *In situ* hybridization on coronal adjacent sections of E22/23 **(A–D)** and E24/25 **(E–H)** with *Tbr2* **(A,E)**, *Pax6* **(B,F)**, *Tbr1* **(C,G)** and *Emx1* **(D,H)** probes. **(I,J)** Schematic representations of the coronal levels corresponding to **(A–D)** or **(E–H)** respectively. *Tbr2* **(A)** apart from the pallial svz and the vz of the VP, was further expressed by postmitotic neurons in the Septum (Se) and the VP mantle (rld) which are both known sources of CR neurons. Note that, the *Tbr1*-expressing subpial stream of CR neurons in **(C)**, expressed also *Tbr2* **(A,I)**. At E24/25, *Tbr1* was still detected subpially in the basal telencephalon **(G)**, though at lower levels than in E22/23. Note the difference in the *Tbr1* expression pattern: at E22/23 *Tbr1* transcripts labeled diffusely the pallial mantle **(C)** however at E24/25 the cp could be clearly detected as a sharply *Tbr1*-expressing layer **(G)**. *Pax6* was expressed within the mantle of the VP-LP (rld) both at E22/23 **(B)** and E24/25 **(F)**, in neurons migrating toward the OB, as well as in a stream emanating from the dorsal striatal vz toward the olfactory tubule (OT). Arrowhead in **(F)** indicates the dorsal part of the (subpallial) septal striatal vz that was characterized by a prominent *Pax6* gradient indicating that the dorsal (subpallial) septum shares the same molecular identity with the dorsal central striatal subdivision. Dotted line in **(J)** marks the subpial migration of *Pax6*-expressing cells from the dorsal striatal subpallium (central or septal), toward the nucleus accumbens (Acb) and the OT. Scale bars 500 μm.

The feline VP was molecularly characterized by the presence of *Tbr2* (with a salt-and-pepper pattern), *Pax6* (strong expression), and *Lhx2* (weak expression) in the vz, which lacked *Emx1*, *Tbr2* (strong expression) and *Emx1* (weak expression, barely visible in Figure 2A) in the svz, and *Tbr1* and *Emx1* in the cortical plate (cp) (Figures 2, 3A–F, 4A–D,I). The analysis of rostral sections revealed that apart from the lateral telencephalic wall (Figures 2A, 3A, 4B and respective schemata) a small region within the medial-ventral (septal) proliferative zones also displayed the VP molecular profile (strong *Pax6* labeling of the weakly *Emx1*-expressing vz; arrowheads in Figures 2A,B,F, Supplementary Figure 5 and respective schemata); this implied that the VP extends across the rostral ventral telencephalon, including the olfactory bulb, from the lateral to the medial wall, as anticipated by the concentric ring topology (Puelles et al., 2019). In caudal planes, posterior to the internal capsule, the *Tbr2*- PThE was continuous with a small *Tbr2* expressing pallial territory, which gradually grew, ultimately extending into the CGE (Figures 3D–F, see also Figures 5B,F,J). The careful examination of adjacent sections revealed that the vz of this compartment apart from *Tbr2*, presented strong *Pax6*-labeling (Figures 5A,E,I), weak *Lhx2* expression (Figure 5K), being otherwise, devoid of *Emx1* signal (Figures 3B,C,5G). We considered this compartment to correspond to the CVP (Ruiz-Reig et al., 2018). The VP expression profile described above at E26/27 was evident from the earliest stage examined, E22/23 (Figure 6). Interestingly, the VP was the only pallial sector that expressed *Tbr2* (with a “salt-and-pepper” pattern; Figures 7A–D and close-up in Figure 6C) at E22/23 (although *Tbr2* labeling of the LP vz could not be ruled out).

**FIGURE 8:**
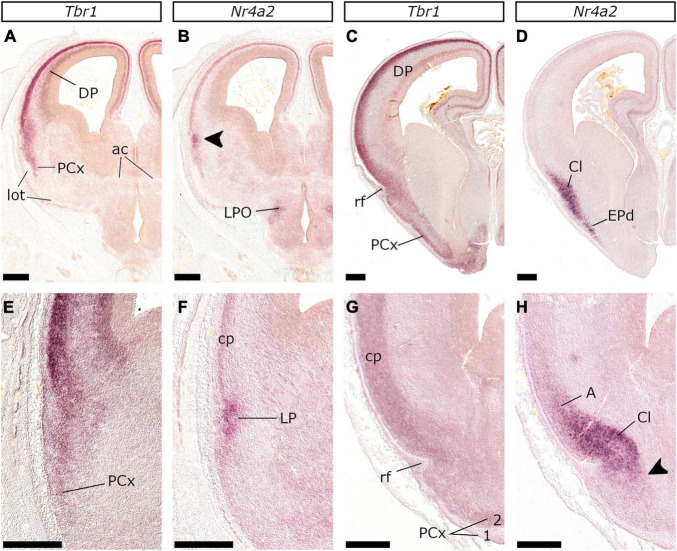
*Nr4a2* is upregulated in the LP mantle around E28/29 facilitating the delineation of the Cl primordium. *In situ* hybridization on coronal sections of the E28/29 **(A,B,E,F)** and the E34/35 **(C,D,G,H)** embryonic telencephalon with *Tbr1* **(A,C,E,G)** or *Nr4a2* **(B,D,F,H)** probes. At E28/29 *Nr4a2* was detected within a subpial focus on the pallial mantle, ventral to the cp at the level of the ac **(B)** or caudal to this **(F)**; there, *Tbr1* expression became more diffuse (compared to the cp) demarcating the anlagen of the *Nr4a2*-expressing claustral complex and the (*Nr4a2*-negative) PCx primordium **(A,E)**. At E34/35, the Cl-primordium could be visualized along the curvature introduced by the rf **(D,H)**. Capital letter A in **(H)** indicates migrating cells (“*Arimatsu*” cells) en-route to the *Tbr1*-expressing isocortical primordium (cp in **G**). The arrowhead in **(H)** indicates the neurons of the dorsal Endopiriform Nucleus (EPd) **(D)** that arise in the claustrum (Cl) domain and migrate ventrally, deep to the PCx layer 2 **(G)**. Scale bars 500 μm.

The VP mantle exhibited (strong) *Tbr1*, (moderate) *Emx1*, and (weak) *Lhx2* expression, associated mainly with the cp. The latter was obvious as a *Tbr1* positive stratum at E22/23 (Figure 6A), E24/25 (Figure 6G), E26/27 (Figure 4I), E28/29 (Figure 8A), and E34/35 (Figure 8C). Furthermore, *Tbr1* expression at E22/23, followed the characteristic pattern described in the mouse (Medina et al., 2004), gradually diminished in the DP (it was unclear however if the medial pallial mantle expressed *Tbr1* at this stage, or if its levels were below the detection limit of our technique; compare the expression of *Tbr1*, *Tbr2*, and *Emx1* in Figures 7A–D). Given the role of *Tbr1* in the neurogenesis of early-born glutamatergic neurons (Hevner et al., 2001), we assumed that in the cat, the first pallial post-mitotic neurons arise around E22/23 in the VP mantle.

#### The Medial Pallium

**FIGURE 9:**
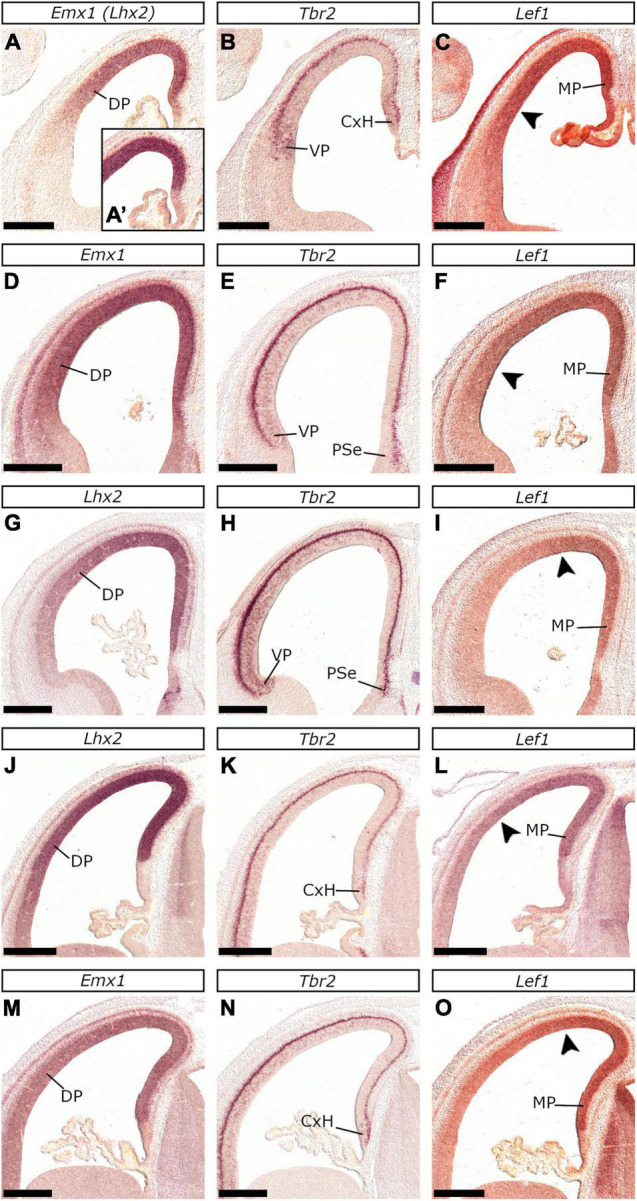
The dynamic expression of *Lef1*, reveals gradual spatiotemporal restriction of the feline MP from E22/23 to E26/27. *In situ* hybridization on coronal sections of the feline embryonic brain at E22/23 **(A–C)**, E24/25 (rostral: **D–F**; caudal: **J–L**) and E26/27 (rostral: **G–I**; caudal: **M–O**) with *Emx1* **(A,D,M)**, *Tbr2* **(B,E,H,K,N)**, *Lef1* **(C,F,I,L,O)** and *Lhx2* **(A′,G,J)** probes. The MP was characterized by *Lef1* **(C,F,L,I,O)**, *Lhx2* **(A′,G,J)** and *Emx1* **(A,D,M)** expression in the vz and *Tbr2* in the svz **(B,E,H,K,N)**. Notably, at E22/23 *Lef1* was detected in a large part of the pallium (arrowhead in **C**) almost up to the (*Emx1*-negative, uniquely *Tbr2*-expressing) vz of the VP **(B)**. Arrowheads in **(F)** and **(L)** indicate the boundary of the *Lef1* domain in the DP at E24/25, which appears restricted in comparison to E22/23 **(C)**. At E26/27 *Lef1* expression was detected in the MP and the medial-most DP sector (arrowheads in **I** and **O**). *Tbr2* at E22/23 was expressed in the vz of the VP (antihem) and the cortical hem (CxH) **(B)**, but at E24/25 and E26/27 its expression domain extended up to the vz of the DP **(E,H,K,N)**. Notably, the MP was the only pallial sector not expressing *Tbr2* in the vz up to E26/27 **(H,N)**. Scale bars 250 μm **(A–C)** and 500 μm **(D–O)**.

**FIGURE 10:**
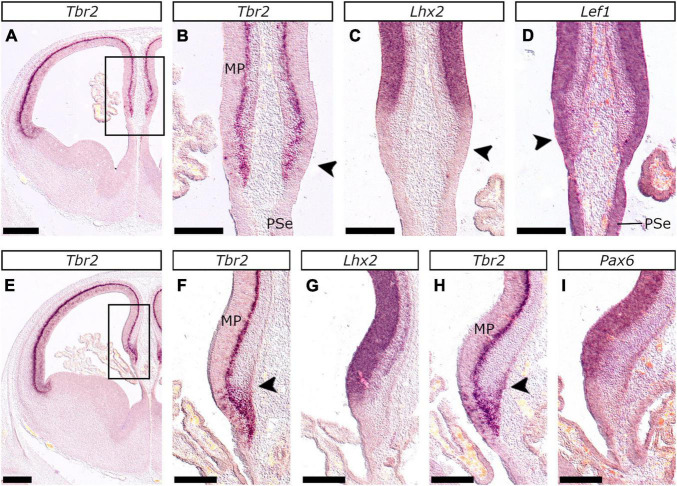
Development of the hippocampal anlage in the E24/25 and E26/27 feline telencephalon. *In situ* hybridization on coronal sections of the feline embryonic pallium at E24/25 **(A–D)** and E26/27 **(E–I)** with *Tbr2* **(A,B,E,F,H)**, *Lhx2* **(C,G)**, *Lef1* **(D)** and *Pax6* **(I)** probes. **(B–D)** and **(F–I)** are close-ups of the boxed areas in **(A)** and **(E)** respectively. The CxH at E22/23 (arrowhead in **B–D**) was characterized by *Tbr2* **(B)** and *Lef1* **(D)** expression in the vz, which lacked however *Lhx2* **(C)**. *Lef1* was further expressed in the PSe **(D)**. *Tbr2* was expressed in the mantle region between the CxH and the abutting MP (arrowhead in **F**) at E26/27; this area (arrowhead in **H**) became wider in more advanced embryos of the same stage and represented the transient subpial neurogenic zone (or secondary matrix). Notably, the CxH did not express *Lhx2* **(G)** or *Pax6* **(I)**. Scale bars 500 μm in **(A,E)** and 250 μm in **(B–D,F–I)**.

At E26/27, *Lef1*, *Lhx2*, *Emx1*, *Tbr2*, and *Pax6* expression characterized the MP proliferative zones, while *Tbr1*, *Lhx2*, *Emx1*, and *Lef1* transcripts appeared in the MP mantle. Our analysis was particularly based on the pattern of *Lef1*, given its selective expression within the MP (Abellán et al., 2014) and its role in hippocampal development (Galceran et al., 2000). At rostral levels of the E26/27 embryonic forebrain, weak *Lef1* expression was observed in the vz of the dorsal-most telencephalic wall and the dorsal aspect of the medial telencephalic wall (arrowheads in Figure 2D). In more caudal planes, the *Lef1*-positive territory covered the entire medial telencephalic wall, expanding to the pallial septum and slightly into the DP (Figures 4C, 9I,O). *Lef1*-expression in the vz of E22/23 embryos expanded far beyond the MP, into the rest of the pallial sectors, almost reaching the VP (with weaker labeling than in the MP though; Figure 9C). At E24/25 however, the *Lef1* expression domain in the DP appeared smaller (Figures 9F,L) in comparison to that of E22/23 (Figure 9C), while in E26/27, *Lef1* covered, apart from the pallial septum, only the MP-abutting DP vz (Figures 9I,O). An interesting feature of the MP at E26/27 was that, in contrast with the other pallial divisions, it was mostly devoid of vz-associated *Tbr2* expression (Figures 10E,F,H; see also Figure 4A). However, the medial most region of the telencephalic wall, just adjacent to the choroid tela, displayed a patchy expression pattern of *Tbr2* (Figures 10F,H) in the vz, which was devoid of *Emx1* (Figure 9M), *Lhx2* (Figure 10G) and *Pax6* (Figure 10I). Apart from E26/27, this was also observed at E22/23 (Figures 9A, A′,B) and E24/25 (Figures 10A-C); based on the genoarchitectonic profile described and its proximity to the choroid plexus, this part was considered to represent the cortical hem (CxH).

#### The Dorsal and Lateral Pallia

Regarding the DP and the LP, there is, to our knowledge, no single marker that exclusively labels their proliferative zones. Differential analysis of several gene expression patterns in the feline pallium revealed, however, that at E26/27 the DP expressed *Emx1*, *Lhx2*, *Pax6*, and *Tbr2* throughout its proliferative zones (Figures 2, 4), and *Tbr1* (data not shown), *Emx1* (Figure 4B), and (weak) *Lhx2* (Figure 4D) in the cp. As already mentioned, the vz of the DP domain that neighbored the MP, showed weak *Lef1* expression. This molecular profile was further used to demarcate the DP in E22/23 and E24/25 embryos (Figures 6, 7, 9). In mice, the LP vz is interposed between the *Emx1/Lhx2*-enriched DP and the *Emx1*-negative VP, and it is characterized by weak *Emx1* and *Lhx2* expression (Puelles et al., 2000; Abellán et al., 2014; Desfilis et al., 2018). In our experiments, however, the LP vz was not clearly demarcated in any of the stages studied as we did not detect sharp boundaries between the expression domains of the aforementioned genes. Based on the Puelles et al. (2016a) approach, however, we identified the LP mantle in E28/29 embryos, as an *Nr4a2*-labeled domain (Figures 8B,F) within the *Tbr1*-expressing region (Figures 8A,E). At E34/35, *Nr4a2*-labeling demarcated the claustral domain (Figures 8D,H), the dorsal endopiriform nucleus (ventralward migrating populations, Dorsal Endopiriform Nucleus (EPd) in Figure 8D and arrowhead in Figure 8H) as well as dorsalward migrating cells entering the isocortex (A in Figure 8H). Notably, *Nr4a2* expression was not detected in the claustral primordium (and in general in the pallium) of E26/27 and earlier embryos. The subpallial mantle however showed weak *Nr4a2* labeling in an area coexpressing *Er81* (Supplementary Figure 2) and other subpallial genes (data not shown).

### Major Compartments of the Subpallium

To delineate molecularly distinct territories within the feline subpallium we studied the expression of *Dlx2*, *Mash1*, *Nkx2-1*, *Er81*, *Pax6*, *Lhx2*, *Lhx6*, *Lhx7*, and *Gad2* that have been used in similar studies in other vertebrates. We analyzed our results according to the developmental ontology (Puelles et al., 2013, 2016b; Watson et al., 2017).

#### The Subpallial Proliferative Zones

##### The Striatum

**FIGURE 11:**
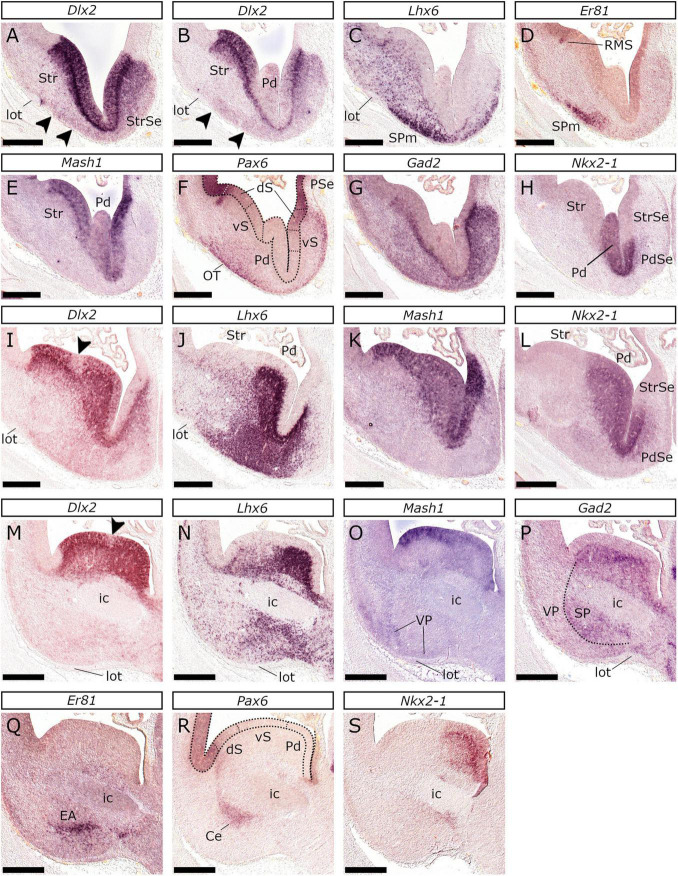
Striatal and pallidal divisions in the subpallium of the E26/27 feline telencephalon. *In situ* hybridization on coronal sections of E26/27 embryonic subpallium with *Dlx2* **(A,B,I,M)**, *Lhx6* **(C,J,N)**, *Er81* **(D,Q)**, *Mash1* **(E,K,O)**, *Pax6* **(F,R)**, *Gad2* **(G,P)** or *Nkx2-1* **(H,L,S)** probes. At rostral levels **(A)** *Dlx2* was highly expressed within the proliferative zones of the SP; *Dlx2* was also expressed in the mantle of the StrSe and weakly at the subpial mantle of the SP (arrowheads), up to the lot. Caudal to this level, the proliferative zones of the SP expressed *Dlx2* **(B)**, *Mash1* **(C),** and *Gad2* **(G)**, while *Nkx2-1* **(H)** delineated the Pd. The proliferative zones of the Str were divided into a dorsal (dS) and a ventral (vS) part; *Pax6* **(F,R)** and *Er81* **(D)** were expressed in the former. In the svz of the dS, a small population expressing *Er81* and *Pax6* represented the primordium of the RMS **(D,F)**. *Nkx2-1* **(H,L)** and *Pax6* **(F)** expression divided the septum in Pd (PdSe), ventral striatal (vS) and dorsal striatal (dS) subdivisions. The mantle of the central subdivision of the SP expressed *Lhx6* **(C)** and *Er81* **(D)**. *Pax6* expressed subpially, demarcated the OT anlage **(F)**. Note that the vz of the rostral aspect of the Pd did not express *Dlx2* **(B)**. The Pd svz was characterized by *Dlx2* **(I,M)**, *Mash1* **(K,O)**, *Nkx2*-*1* **(L,S)** and *Lhx6* **(J,N)** expression; in contrast, the Str svz expressed only *Dlx2* **(I,M)**. *Dlx2* was not detected in the region between the central subdivisions of the Str and the Pd (arrowhead in **I,M**) that corresponds to the pLGE4-pMGE1 regions. At levels around the ic **(M–S)** weak expression of *Mash1* was observed in the VP along the lot **(O)**. The dotted line in **(P)** indicates the PSB. *Pax6* was expressed in the crescent-shaped Ce primordium **(R)**, while *Er81* demarcated cell populations of the EA **(Q)** related to the GP (“sublenticular”). Scale bars 500 μm.

The vz of the feline striatal anlage was molecularly characterized by *Dlx2* (Figures 4E, 11A,B,I,M), *Mash1* (Figures 4G, 11E,K,O) and *Lhx2* (Figure 4D) expression but not by *Nkx2-1* (Figures 4H, 11H,L,S). *Pax6* was also detected in the striatal vz, but with a lateral-high to a medial-low gradient of expression (Figures 11F,R; present also on the septal striatum, arrowhead in Figure 7F). In the striatal svz, *Dlx2* (Figures 4E, 11A,B,I,M) and *Gad2* (Figures 4K, 11G,P) were detected. *Lhx6* expression was also observed in a “salt and pepper” pattern (Figures 4F, 11J,N) in the tangentially migrating, pallidal-derived, interneurons, en route to the pallium (Liodis et al., 2007). The dorsal-most aspect of the feline striatal svz was characterized by a strong focal expression of *Pax6* (Figure 11F) and *Er81* (Figure 11D). Interestingly, *Er81* was also weakly expressed in a small part of the dorsal-most striatal vz (Figure 11D) overlying the strongly *Er81*/*Pax6*-positive svz area; thus, the feline striatal anlage proliferative zones were divided into two subdivisions, a dorsal and a ventral. Ventrally, the striatal vz showed a spot devoid of *Dlx2*, but with high *Mash1* and *Lhx2* expression (compare Figures 4D,E,G, 11I,K,M,O). In mice this area is associated with the *Nkx6-2*-expressing, intereminential sulcus region shared between the progenitor domains pLGE4 and pMGE1 (Flames et al., 2007); accordingly, the pLGE4 in the cat is an *Lhx2*-positive (Figure 4D), *Mash1*-positive, but *Dlx2*-negative domain within the ventral striatal anlage. The striatal subdivision extended throughout the rostrocaudal axis, from the lateral-ventral telencephalic wall rostrally (Figure 1A), to the dorsal tip of the caudal-most end of the amygdaloid division caudally (Figures 1I, 3J–L). However, as in mice (Flames et al., 2007), the *Dlx2*-negative pLGE4 domain did not extend caudally to the level of the internal capsule (compare Figures 3J,K, 11M). Notably, in caudal levels (Figure 5I), the subpallial svz showed *Pax6* expression both in its lateral (VP-abutting) and medial aspect (CVP-abutting), suggesting that the AStr bears the dorsal striatal molecular profile.

##### The Pallidum

The vz of the pallidal anlage featured strong *Mash1* (Figures 4G, 11K), *Dlx2* (Figures 4E, 11I), and *Nkx2-1* (Figures 4H, 11L) labeling, being however is devoid of *Lhx2* (Figure 4D) and *Pax6* (Figures 11F,R) expression; the svz expressed *Lhx6* (Figures 11J,N), *Dlx2* (Figures 4E, 11I), *Nkx2-1* (Figures 11L,S), *Gad2* (Figures 4K, 11P), and *Mash1* (Figures 11K,O, particularly the svz2, in contrast with the *Mash1*-negative/*Dlx2*-positive svz2 of the striatal anlage; shown in Figure 4G). Interestingly, the vz of the rostral-most aspect of the pallidal division (Figure 11B) lacked *Dlx2* expression (*Dlx2* was detected in the svz); *Mash1* and *Nkx2-1* expression were, however, detected (Figures 11E,H). Coronal planes caudal to the internal capsule demonstrated the shrinkage of the pallidal domain as shown by the expression of *Dlx2* and *Nkx2-1* (Figures 3J,J′) that spatially coincided with the expansion of the CVP.

##### The Preoptic and the Diagonal Area

**FIGURE 12:**
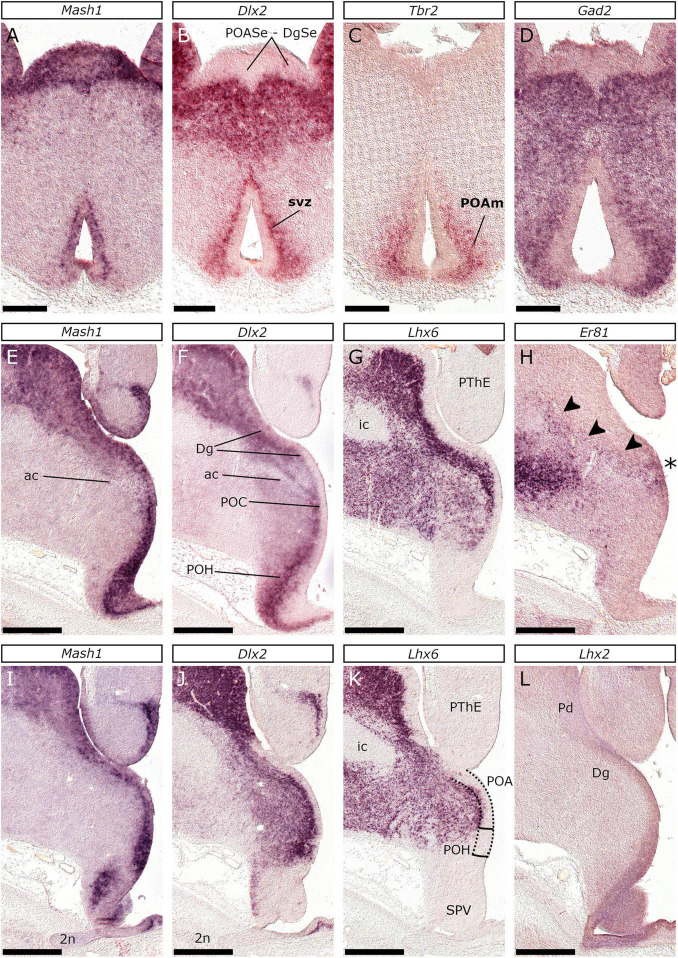
The diagonal and the preoptic area at E26/27 are characterized by a lack of *Dlx2* expression in the vz. *In situ* hybridization on coronal sections of the E26/27 embryonic subpallium with *Mash1* **(A,E,I)**, *Dlx2* **(B,F,J)**, *Tbr2* **(C)**, *Gad2* **(D)**, *Lhx6* **(G,K)**, *Er81* **(H)** or *Lhx2* **(L)** probes. The POA was characterized by *Mash1* expression in the vz **(A,E,I)** and lack of *Dlx2* **(B,F,J)** which was nevertheless expressed in the POA svz (svz in **B**); *Tbr2* **(C),** and *Gad2* **(D)** were detected in the POA mantle (POAm in **C**). *Lhx6* was expressed in the dorsal-most area of the POA **(G,K)**, which is associated with the ac (POC in **F**). Notably, *Lhx6* expression reduced at the hypothalamus-abutting POA (POH). *Lhx2* was detected within the vz of the POA and the Dg **(L)**; around this level, *Er81* expression was detected in the vz between the Dg and the POA (asterisk in **H**). Arrowheads in **(H)** indicate neurons migrating from the Dg to the GP. Scale bars 250 μm **(A–D)** or 500 μm **(E–L)**.

The feline POA was rostrally identified, as the wall lining the rostral-most recess of the third ventricle (Figures 12A–D). Interestingly, the POA vz expressed *Mash1* (Figure 12A) and *Nkx2-1* (Figure 4H), lacking, however, *Dlx2* expression (Figure 12B). Nevertheless, *Dlx2* labeled the POA svz (and mantle), which uniquely expressed *Tbr2* (Figure 12C), along with *Gad2* (Figure 12D). Notably, at this level, the POA svz/mantle lacked *Lhx6* expression (Figure 4F). Coronal levels around the anterior commissure further revealed that the POA (or POC, according to García-López et al., 2008) vz expressed *Mash1* (Figure 12E) and *Nkx2-1* (Figure 4H), but not *Dlx2* (Figure 12F). At the ivf level, *Er81* labeled a small part of the vz (asterisk in Figure 12H) which also expressed *Mash1* (Figure 12E) but not *Dlx2* (Figure 12F); this area is closely associated with the anterior commissure, in the “turn” between the evaginated and the non-evaginated telencephalic compartments (hp2 and hp1 respectively). Furthermore, a thin *Er81*-expressing streak of cells emanating from this region and extending into the subpallial mantle was detected (Figure 12H arrowheads). Given the transcriptional codes demonstrated in the murine telencephalon (Flames et al., 2007), previous data on POC and the Dg (García-López et al., 2008; Puelles et al., 2016b) and the fact that *Er81*-lineage GP neurons arise from the latter (Nóbrega-Pereira et al., 2010) we believe that this ventricular *Er81*-expressing area represented the border between the Dg (dorsally) and the POA (or POC, ventrally). *Lhx6* labeled the svz of the Dg and the dorsal-most domain of the POA (Figures 12G,K). Notably, the ventral-most (hypothalamus abutting) preoptic svz remained *Lhx6*-negative (compare Figures 12F,J respectively to Figures 12G,K). Furthermore, *Lhx2* was weakly expressed in the vz of the preoptic and the diagonal area, in contrast with the *Lhx2*-negative pallidal anlage (Figure 12L).

##### The Septum

The septum encompasses the septal areal subdivisions of the striatal, pallidal, diagonal, and preoptic domains rather than being a subpallial radial domain itself (Puelles et al., 2013, 2016b). For descriptive reasons, however, we refer to the septum separately, out of the context of the radial domains. The feline striatal septum appeared in the rostral sections, anterior to the rise of the pallidal domain (Figure 11A), and, alike the central striatal division, was characterized by *Dlx2*, *Pax6*, and *Er81* expression in the proliferative zones. The pallidal septal vz was almost devoid of *Dlx2* labeling (Figures 11B,I), however, it showed strong *Nkx2-1* (Figure 11L) and *Mash1* (Figure 11K) expression. The corresponding svz was *Dlx2* (Figure 11I), *Lhx6* (Figure 11J), and *Gad2* (data not shown) positive. Notably, the pallidal molecular profile characterized the ventral half of the septal proliferative zones; the vz of the dorsal septal part (ventral to the pallial septum) was characterized by *Mash1* (Figure 11K), but not *Nkx2-1* expression (Figure 11L). The underlying svz showed high *Dlx2*-expression (Figure 11I), being, however, *Lhx6* negative (Figure 11J). We thus propose that the dorsal-most (pallial-septum abutting) septal part is striatal in nature. The preoptic septal subdivision is closely associated with the POA; however, apart from lack of *Dlx2* expression, it did not differ molecularly from its pallidal counterpart (Figures 12A–D).

#### The Subpallial Mantle

The feline subpallial mantle was in general characterized by *Gad2* (Figures 4K, 11P) expression. At rostral levels, *Dlx2* was expressed in the subpallial septal mantle and, at low levels in the subpial territory of the paraseptal and central striatal subdivisions (arrowheads in Figures 11A,B); these subdivisions presented further a thin *Pax6* expressing band below the pial surface (Figures 7F, 11F). In previous studies in mice (and chicken; Puelles et al., 2000) these territories were considered to represent the anlagen of the olfactory tubercle (OT) (adjacent to the lot) and the Acb (around the medial-ventral portion of the mantle). In the pallidal territory, strong *Lhx6* expression of the *Dlx2*/*Pax6*-positive subpial stream of cells (Figure 11C) was observed, with a subpopulation expressing *Er81* (Figure 11D). Caudally, high *Lhx6* expression was detected in the pallidal mantle; notably the subpial area of the subpallial mantle (striatal or pallidal) presented highly *Lhx6* expressing cells (Figures 4F, 11J). The pallidal mantle contained the *Nkx2-1* expressing GP anlage, that was also labeled by *Gad2*, *Lhx6*, *Lhx7*, and *Er81*, best demonstrated around the ivf plane (Figures 4H,K,F,J,L); the expression of these markers was also observed in the compartments of the EA. Interestingly, coronal planes just rostral to the ivf and the *Tbr2*-expressing POA revealed a *Tbr1*/*Pax6* expressing domain located between the *Gad2*-labeled central, paraseptal, and septal subdivisions of the pallidal mantle (Figures 6A,B,G,H), that we considered representing the anlagen of the DB nuclei (Puelles et al., 2000). Careful examination of E22/23 sections identified a stream of cells weakly expressing *Tbr1* (arrowheads in Figure 6D) and *Pax6* (arrowheads in Figure 6F) that extended between the DB and the OT, presumably reaching the VP. This was thought to represent septal-derived glutamatergic neurons en-route to the OT (Ceci et al., 2012). We propose that the *Pax6*/*Tbr1* expressing region corresponds to the mantle of the Dg sector. Coronal planes around the level of the internal capsule showed that the *Gad2* expressing subpallial mantle was surrounded by the VP, which weakly expressed *Mash1* (compare Figures 11O,P). Additionally, *Pax6* expression in the subpallial mantle was used to identify in the cat the crescent-shaped Central amygdalar nucleus (Ce) (Figure 11R) and the anterior amygdala (AA) Figure 5A), as described in mice (Bupesh et al., 2011). Moreover, the *Er81*/*Lhx6* expressing population represented in the cat the extended (sublenticular) amygdala (EA, Figure 11Q) as described in the mouse (García-López et al., 2008). Caudally, the subpallial mantle reduced (compare *Emx1*/*Dlx2* expression in Figure 3), at the expense of the pallial amygdala. As mentioned before, the caudal-most area of the basal telencephalon is molecularly ventropallial (VAP), except for its dorsal-most *Dlx2*/*Mash1*-expressing tip (AStr; Figure 3L). Notably, the *Tbr2*-negative core (Figure 3F) of the VAP, showed *Lhx6* expression (Figure 3I), corresponding to the migrating interneurons of the caudal migratory stream (CMS) (Touzot et al., 2016).

### Dynamic Gene Expression in the Olfactory Bulb and the Amygdala

The olfactory bulb and the amygdala lie at the rostral and caudal poles of the embryonic telencephalon respectively. As both the pallium and the subpallium contribute to their formation, we chose to study these entities out of the strict pallial or subpallial context. To this end, we followed the dynamic *Tbr2* expression, in combination with *Pax6*, *Tbr1*, *Er81*, and *Gad2* to approach ontogenesis of the olfactory bulb. To study the amygdala, we primarily utilized the dynamic *Tbr2, Emx1*, and *Pax6* expression profiles, as well as *Lhx2* and *Tbr1*.

#### The Olfactory Bulb

**FIGURE 13:**
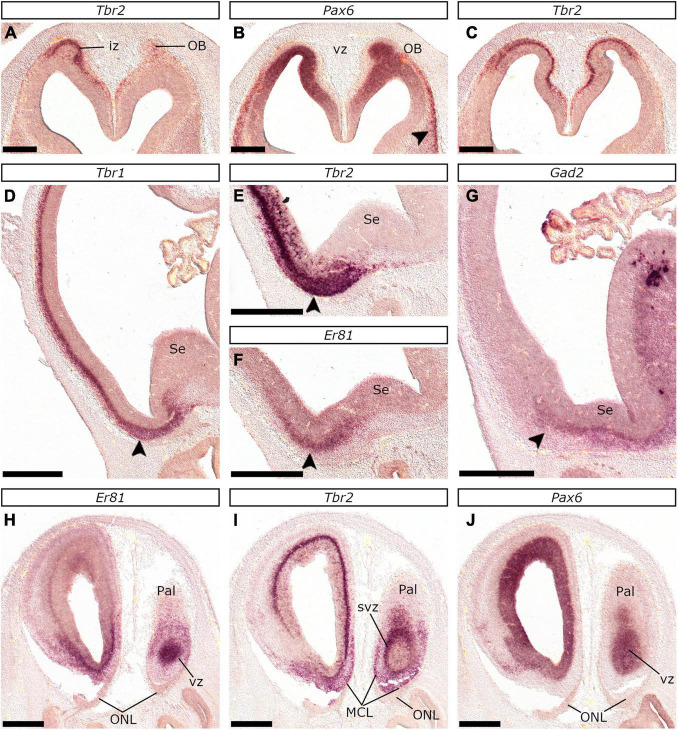
Development and neurogenesis in the feline OB between E22/23 and E26/27. *In situ* hybridization on coronal sections at E22/23 **(A–C)** and E26/27 **(H–J)**, or sagittal sections at E24/25 **(D–G)** of embryonic brains with *Tbr2* **(A,C,E,I)**, *Pax6* **(B,J)**, *Tbr1* **(D)**, *Er81* **(F,H)**, or *Gad2* **(G)** probes. At E22/23, the OB could be identified as an evagination of the rostral-most pallial region, that expressed *Pax6* in the vz **(B)** and *Tbr2* in the iz **(A,C)**. Arrowhead in **(B)** points to *Pax6*-expressing neurons of the olfactory cortex. Sagittal sections at E24/25 revealed considerable thickening of the iz, which highly expressed *Tbr1* **(D)** and *Tbr2* **(E)**. *Er81* **(F)** and *Gad2* **(G)** were expressed in the OB primordium indicating settlement of SP-derived interneurons. Arrowheads in **(D–G)** indicate the OB primordium. Coronal sections at E26/27 showed that the vz of the OB expressed *Pax6* **(J)** along with *Er81* **(H)**. Furthermore, the iz was considerably thickened, while *Tbr2* expression was restricted in the basal aspect of the vz (svz in **I**) and the subpial area; the latter was in close contact with the ONL and represented the incipient MCL. Scale bars 250 μm **(A–C)** or 500 μm **(D–J)**.

The OB can be visualized as an evagination of the rostral-most area of the telencephalic wall, even in E22/23 embryos (Figures 13A–C). At this early stage, the OB consisted of two layers (like in rodents Hinds, 1968; Imamura and Greer, 2013): the *Pax6*-labeled ventricular zone (vz) (Figure 13B) and the *Tbr2*-expressing mantle (or intermediate zone, iz; Figures 13A,C). At E24/25 the vz expressed *Pax6* (data not shown) similarly to E22/23; moreover, the *Tbr2*-labeled iz thickened (compare Figures 13B,E) and showed expression of *Tbr1* (Figure 13D), *Gad2* (Figure 13G), and *Er81* (Figure 13F). Quite interestingly at E26/27, the OB vz, apart from *Pax6* (Figure 13J), expressed high levels of *Er81* (Figure 13H). At this stage, in the considerably thickened iz, *Tbr2* was still expressed, yet with a distinct pattern. More specifically, two cell layers of high *Tbr2* expression were detected: one demarcating the limit between the vz and the iz, and a second beneath the pia, in close contact with the olfactory nerve layer (ONL; Figure 13I and Supplementary Figures 3A, B, D). The former, further expressed *Tbr1* (evident in E28/29, Supplementary Figure 3C) and was considered to represent the svz (or subependymal layer) of the OB. The subpial *Tbr2*-expressing stratum was evident at E28/29 and E34/35 and was considered to represent the mitral cell layer (MCL) primordium. GABAergic (*Gad2*-expressing) and dopaminergic (*Er81*-expressing) juxtaglomerular interneurons settle at the OB primordium around E24/25. Interestingly, within retrobulbar planes, we were able to locate strongly *Er81*-expressing cells, migrating along the nervus terminalis, from the vomeronasal organ toward the OB anlage (Supplementary Figure 4). These were considered to represent GnRH-producing neurons that populate the basal telencephalon (Tarozzo et al., 1995; Døving and Trotier, 1998).

#### The Amygdala

**FIGURE 14:**
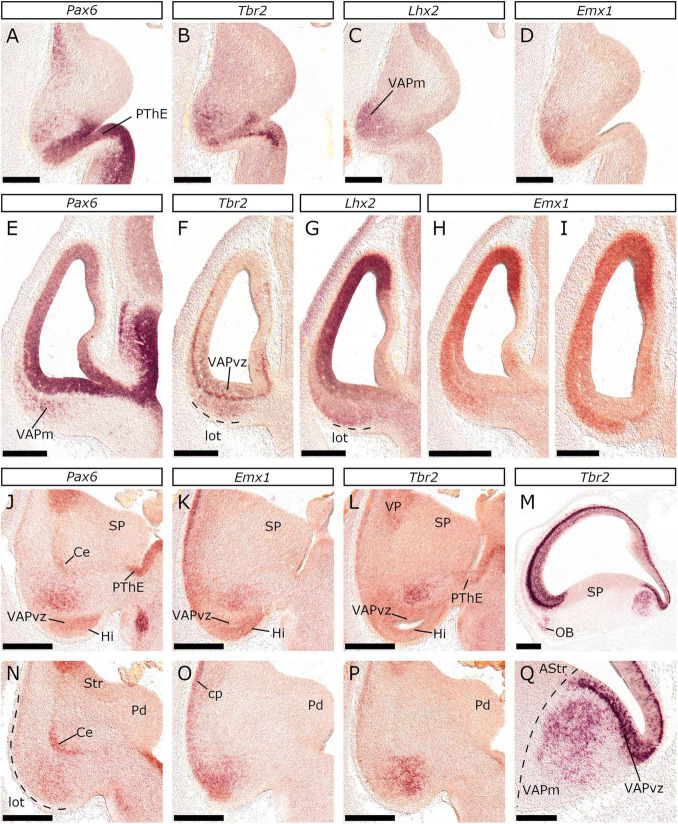
Generation of the first amygdalar nuclei between E22 and E25. *In situ* hybridization on coronal or horizontal sections of E22/23 **(A–I)** or E24/25 **(J,Q)** embryonic brains with *Pax6* **(A,E,J,N)**, *Tbr2* **(B,F,L,M,P,Q)**, *Lhx2* **(C,G)** and *Emx1* **(D,H,I,K,O)** probes. Sections at the horizontal or the coronal plane revealed at E22/23 diffuse *Lhx2* **(C,G)** and *Emx1* **(D,H,I)** expression in the VAPm; *Pax6* **(A,C)** and *Tbr2* **(B,E)** were detected with a “salt-and-pepper” pattern. Dashed lines in **(F,G)** demarcate the lot. **(A–D)** and **(E–H)** are serial horizontal or coronal planes respectively. The plane of section in **(I)** is caudal to that of (H). At E24/25 gene expression patterns were focal (unlike the diffuse patterns at E22/23), resembling an incipient (yet apparent) nuclear organization **(J,Q)**. Sections in **(J–L)** and **(N–P)** are dorsal (deep) or ventral (superficial) horizontal planes respectively. A dashed line in **(N)** indicates the lot. **(Q)** is a close-up of the lower-right area of **(M)**, demonstrating the migration of *Tbr2* positive neurons from the VAP proliferative zones to the VAPm. Note that in **(Q)**, *Tbr2-*expressing cells are more dispersed in the superficial area, than in the deep (close to the svz). The dashed line in **(Q)** indicates the PSB. Scale bars 250 μm **(I,Q)** and 500 μm in all other figures.

To gain an insight into the organization of the feline pallial amygdala, we studied the dynamic *Pax6*, *Emx1*, and *Tbr2* expression patterns at E22/23, E24/25, and E26/27, using not only coronal but also sagittal and horizontal sections. At E22/23, diffuse *Emx1* expression (Figures 14D,H,I) was detected within the VAP mantle (VAPm). *Lhx2* further labeled the VAPm (Figures 14C,G), which also expressed *Tbr2* (Figures 14B,F) and *Pax6* (Figures 14A,E) in a “salt-and-pepper” pattern. At E24/25, *Pax6*, *Emx1* and *Tbr2* expression profiles within the VAPm changed from diffuse to focal. More specifically around the level of the PThE, *Pax6*, *Emx1*, and *Tbr2* expressing cells gathered to form foci, positioned between the Ce primordium and the vz of the VAP (Figures 14J,K). At horizontal levels ventral to this, *Pax6* expression was reduced (Figure 14N) and *Emx1* labeled cells (Figure 14O) gathered more superficially, near the lot. *Tbr2* expressing cells (Figure 14P) on the other hand, were detected dispersed, more medially, with a pattern almost complementary to that of *Emx1* (compare Figures 14O,P). In sagittal sections (Figures 14M,Q), the *Tbr2* expressing domain could be divided into a dorsal/deep domain, in close association with the vz of the VAP, and a ventral territory extending superficially; *Tbr2* expressing cells appeared tightly assembled in the former and dispersed in the latter (Figure 14Q).

At E26/27, coronal sections at levels caudal to the ic revealed diffuse *Pax6* expression (Figure 5A) in the transition region between the caudal Ce and the AA. Weak *Lhx2* expression could be further observed in a cell population emanating from the VP and extending deep to the AA (Figure 5C). At this plane, *Tbr2* expression was observed in a small superficial part of the VAPm that also showed *Pax6* expression (compare Figures 5B,C). *Tbr1* expression extended all along the lot up to the bed nucleus of the accessory olfactory tract (BAOT) primordium (Figure 5D). Caudal to this level, almost identical *Pax6* and *Tbr2* expression patterns were observed, with a dorsal and a ventral domain (Figures 5E,F) surrounding the *Emx1*-expressing territory (Figure 5G). Given that *Emx1* is expressed in the anlagen of the BL and the PLCo (Puelles et al., 2000; Medina et al., 2004), whereas *Pax6* within the BM (Puelles et al., 2000) and the Me (Bupesh et al., 2011; Duan et al., 2013), we identified the dorsal *Pax6*/*Tbr2* expressing focus as the BM anlage. Moreover, we considered the superficial *Tbr2*-expressing domain to represent superficial corticoid nuclei (ACo), given (1) its position between the *Emx1* positive BL/PLCo and the *Emx1*/*Tbr1* negative Me primordia and (2) the fact that both BMA and ACo are products of the anterior amygdalar radial unit (Garcia-Calero and Puelles, 2021). On the other hand, it could belong to the MePV, given that its core has been reported to express both *Pax6* and *Tbr2* (Ruiz-Reig et al., 2018). Careful examination of *Lhx6* preparations at the same coronal level identified the NLOT, as a focus almost devoid of subpallial-derived migrating interneurons (Figure 3G and schema). At levels immediately posterior to the previous, the BM anlage (*Tbr2*/*Pax6*-expressing, dorsal domain) continued to extend caudally (Figures 5I,J), while the rest of the VAPm expressed *Emx1* (data not shown) and *Tbr1* (Figure 5L). Finally, at the caudal-most planes of the VAPm, neither *Tbr2* (Figure 3E) nor *Pax6* (data not shown) expression was detected.

## Discussion

Comparative gene expression analysis and molecular fate mapping have revealed the principles underlying vertebrate brain design, leading to the emergence of the field of evolutionary developmental neurobiology (neuro-evo-devo, Striedter, 2007). Gene expression studies, however, not only unravel brain genoarchitecture, described by the prosomeric model and the relevant ontology, but also pinpoint differences, that may affect the brain morphology and function even within a small taxonomic group, like mammals (Molnár et al., 2006; Rakic, 2009; Martínez-Cerdeño et al., 2018). In this work, we have analyzed the genoarchitectonic compartmentalization of the developing feline telencephalon, studying the expression of genes, the orthologs of which have been successfully used in other vertebrates (e.g., Puelles et al., 2000; Flames et al., 2007; Abellán et al., 2014; Desfilis et al., 2018).

In the developing feline pallium, the expression of *Pax6*, *Tbr2*, and *Tbr1* marks radial glia, intermediate progenitors, and post-mitotic neurons, or vz → svz (already present at E22/23) → mantle, respectively. Additionally, neurogenesis is spatiotemporally regulated: the VP mantle is the first to appear, while the MP mantle is the last; as in mice, a gradient of *Tbr1* expression with high levels in the VP mantle progressively decreasing toward the MP mantle was observed (Bulfone et al., 1995; Medina et al., 2004). The pallial mantle appeared at E22/23 as *Tbr1* expressing cells aggregate forming a thin lamina; the cp however was clearly detected one day later at E24/25 as a *Tbr1*-expressing layer. At this stage, lamination within the pallial mantle, correlated with the *Tbr1* expression in the cp and the (*Tbr1*-positive) subplate (sp)/intermediate zone (iz); these are clearly detected as distinct layers in the lateral telencephalic wall and merge gradually to form a single layer in the MP. In addition, the DP cp is clearly separated from the underlying sp/iz at E24/25, while the MP cp at E28/29 (Figure 8A). The dynamic expression pattern of *Tbr2* in the vz further supports a sequential mode of pallial neurogenesis. In mice, *Tbr2* upregulation in the vz reflects an increase in the production of intermediate progenitors at the peak of neurogenesis. Interestingly, in the feline embryo, at E22/23, the only pallial domain expressing *Tbr2* is the VP vz. At E24/25 and E26/27 however, *Tbr2* expression domain gradually expands into the vz of the DP. Accordingly, the vz of the MP is the last to upregulate *Tbr2* expression around E30. Previous research in cats has demonstrated that subplate cells appear around E24, while layer VI neurons of the visual cortex later, at E31 (Luskin and Shatz, 1985a,b). Moreover, in a recent study (Glatzle et al., 2017) using Tbr1 as a marker, E30 was identified as the stage when deep-layer neurogenesis in cats commences. These results, however, are not in discrepancy with our study, given the heterochrony in the generation of the cp across the various pallial sectors. Indeed, the primary visual cortex (Luskin and Shatz, 1985a,b), along with the dorsal-lateral telencephalic region that was studied by Glatzle et al. (2017), are considered dorsal pallial derivatives; it remains also unclear if earlier stages were screened in the latter study. In mice, Cajal-Retzius (CR) neurons are generated between E10.5 and E12.5 and express, among other factors, *Tbr1* (Hevner et al., 2003; Yoshida et al., 2006; Tissir et al., 2009). We show that in the feline pallium CR neurons first appear as a *Tbr1*-expressing subpial stream, in the basal telencephalon of E22/23 embryos. We also show that CR neurons also express *Tbr2* (Figure 7A). Interestingly at rostral levels, *Tbr2* is also highly expressed in the mantle of the septum and the VP - both regions are major sources for CR neurons in mice (Bielle et al., 2005). As of E24/25, subpial expression of *Tbr1* and *Tbr2* in the basal telencephalon gradually decreases, suggesting that CR generation peaks around E22/23.

At E26/27 the proliferative zones of the VP formed a C-shaped ring, an observation following the concentric ring pallial model (Puelles et al., 2019). The identification of the vz and the svz of the VP was primarily based on the analysis of *Tbr2*, *Pax6*, and *Emx1* expression. Previous studies in mice have shown that the VP vz and svz express *Dbx1*, *Sfrp2*, *Fgf15*, *Lhx9*, and *Gdf10* (Puelles et al., 2000; Kim et al., 2001; Medina et al., 2004; García-López et al., 2008; Ruiz-Reig et al., 2018); these, however, are detected within distinct subregions of the VP, hence, we did not use them in this study. Interestingly, *Emx1* is not expressed in the vz of the VP; this observation led to the updating of the pallial model from tripartite to tetrapartite (Smith-Fernandez et al., 1998; Puelles et al., 2000). *Pax6* is strongly expressed in the vz of the VP (considered to be the antihem), regulating the expression of a battery of genes essential for the formation of the PSB (Kim et al., 2001; Stenman et al., 2003b; Tuoc and Stoykova, 2008; Carney et al., 2009). *Lhx2* on the other hand, antagonizes *Pax6*, suppressing the antihem fate (Godbole et al., 2017); *Lhx2* is, thus, weakly expressed in the vz of the VP. Our results from the analysis of these genes were in accordance with previous findings in mice. Therefore, based on the above, as well as in the observation that *Tbr2* is expressed in the antihem as early as E22/23, we mapped the proliferative zones of the VP (Schemata in Figures 2–4): (1) In the retrobulbar septal region; (2) In the lateral-ventral telencephalic wall; (3) In the caudal-most aspect of the basal telencephalon (vz of the VAP); (4) Continuous with the PThE, in the medial aspect of the basal telencephalic bulge (CVP).

In the updated prosomeric model, the LP has been redefined to include the claustroinsular complex that exclusively expresses *Nr4a2* (Puelles, 2014; Puelles et al., 2016a; Watson and Puelles, 2017). Our results show that in the domestic cat, *Nr4a2* is expressed in the mantle of the LP after E28/29. Notably, this territory is located subpially between the *Tbr1*-expressing cp (dorsally) and the PCx primordium that is more diffusely labeled by *Tbr1* (ventrally). Around E34/35, *Nr4a2* expression marked, apart from the claustroinsular primordium, the migration route of Cl-derived cells toward the isocortex (“*Arimatsu*” cells) or the EPd (Arimatsu et al., 2009; Puelles, 2014). We did not detect *Nr4a2* in LP at earlier stages, however, we observed expression of *Tbr2* and *Pax6* in the LP mantle at the ventral-most border of the *Tbr1*-expressing cp in rostral (yet retrobulbar) pallial fields of E22/23 and E24/25 embryos (rld in Figure 7). This domain could represent the Cl primordium; however, this hypothesis cannot be supported as we did not detect *Tbr2*/*Pax6* expression at caudal levels.

In mice, the MP is strongly associated with the expression of the *Wnt* effector, *Lef1* (Behrens et al., 1996; Galceran et al., 2000). We also observed a dynamic expression pattern of the feline *Lef1* ortholog. At E22/23, the MP primordium covers a large part of the pallium, in later stages, however, DP develops and MP occupies a relatively smaller part. More specifically, at E22/23 *Lef1* expression domain extends throughout the pallial anlage, except for the vz of the VP (antihem) that expresses *Pax6* known to downregulate Wnt signaling (Chou and Tole, 2019). At later stages, however (E24/25 – E26/27), the *Lef1* expression domain is gradually restricted to the vz of the MP and the MP-abutting DP. This dynamic pattern of *Lef1* is also evident in the developing mouse pallium (Abellán et al., 2014, Allen Brain database). Given that MP is the hippocampal primordium, we further propose that, in terms of hippocampal development, the E26/27 feline MP corresponds to the E14.5 murine MP. Even at E22/23, the vz and the svz of the feline CxH expressed *Tbr2*, as already described for mice (Sugiyama et al., 2013). At E26/27, the medial-most (CxH-abutting) mantle of the MP thickened, while in the pial surface and the “mantle” corresponding to the CxH, a *Tbr2* population was detected that appeared expanded in older embryos within the same group. This population may represent *Tbr2*-expressing CR cells of the transient subpial neurogenic zone (or hippocampal secondary matrix) identified at E14.5 in mice (Li et al., 2009; Hodge et al., 2013; Urbán and Guillemot, 2014).

Our study shows that subpallial organization is conserved at least between cats, mice (Flames et al., 2007; García-López et al., 2008) and chicken (Abellán and Medina, 2009; Bardet et al., 2010). This was revealed by the analysis of the expression profiles of genes that delineate striatal, pallidal, diagonal, and preoptic divisions. The striatal domain is shown to parcellate in a dorsal and a ventral compartment, identified by *Pax6* and *Er81* within the vz of the former. The dorsal striatum is, in rostral levels, further characterized by focal expression of *Pax6* and *Er81* in the svz. This domain was considered to represent the rostral migratory stream primordium supplying the OB with *Pax6*/*Er81* -expressing periglomerular neurons. This cell population has been shown to express *Er81* in mice (Stenman et al., 2003a; Long et al., 2007; Figure 1A of Carney et al., 2009; Figure 4A of Rash et al., 2013). Additionally Flames et al. (2007), defined the pLGE1 as the vz region overlying the svz *Er81* focus (see in Flames et al., 2007 their Figure 2D). On the other hand, the same authors identified this focal *Pax6* expressing domain dorsal to pLGE1 (Flames et al., 2007 their Figure 2B) ascribing it to the pallium. Having studied *Er81* and *Pax6* on consecutive sections along the rostrocaudal axis we propose that it is the same population that expresses *Er81* and *Pax6*.

Previous studies in mice have shown that at E12.5 and E13.5, a small vz domain located at the border between the ventral Str (pLGE4) and the dorsal Pd (pMGE1), lacks the expression of *Dlx2* but expresses *Nkx6-2* (Flames et al., 2007). In our experiments, the vz of the rostral-most region of the Pd shows high *Nkx2-1* and *Mash1* expression but lacks *Dlx2*; this indicates that pMGE1 expands to this rostral area. We did not detect *Lhx6* or *Lhx7* expression in the vz of the Pd; thus, we were not able to delineate the rest of the Pd progenitor domains described previously in mice (Flames et al., 2007). The POA in felids can be divided into a dorsal area that is associated with the ac, and a ventral that abuts the hypothalamus, as already described for mice (Abellán et al., 2010). An interesting observation is the identification of a subset of *Tbr2* expressing cells within the *Gad2*-expressing POA mantle. These cells may relate to the POA-native expression of pallial markers *Dbx1* or *Lhx5* (Flames et al., 2007; Gelman et al., 2009; Abellán et al., 2010), or may have migrated from extratelencephalic regions, like the PThE or the telencephalon-opto-hypothalamic domain (Morales et al., 2021). This observation reflects the vast diversity of cellular phenotypes (e.g., cholinergic, GABAergic, glutamatergic, nitrergic or even dual-phenotype Glu-GABAergic neurons, as well as oligodendrocytes) produced by the POA (Kocsis et al., 2003; Ottem et al., 2004; Abellán et al., 2010; Medina and Abellán, 2012). Furthermore, our results suggest that the POA proliferative zones include a subventricular zone, although its existence in mice has been disputed (Gelman et al., 2009). This hypothesis is supported by the detection of a *Dlx2*-expressing streak of cells forming a line abutting the basal aspect of the POA vz (*Mash1*-expressing) and the POA mantle (*Gad2*-expressing).

In cats, the primordium of the OB was evident at E22/23, as an evagination of the rostral-most telencephalic domain; at this stage, *Pax6* and *Tbr2* labeled the vz and the iz, respectively. In mice evagination does not commence prior to E12 (Hinds, 1968); the OB anlage expresses *Pax6*, *Tbr2*, and *Tbr1* as of E11 (Imamura and Greer, 2013). Given that the first mitral cells in mice are generated *via* the non-canonical (*Pax6* → *Tbr1* → *Tbr2*) neurogenic cascade (Imamura and Greer, 2013), we studied *Tbr2* at E22/23, instead of *Tbr1*, to label the nascent projection neurons. *Tbr1* expression was nevertheless detected at E24/25, marking the whole extent of the iz. At E26/27, the nascent MCL appears at the ventral-lateral aspect of the OB as a thin subpial layer expressing *Tbr2*, in close contact with the ONL.

Although in mice the svz of the OB does not express *Tbr2* (Imamura and Greer, 2013), we observed a *Tbr2* positive layer lining the basal aspect of the OB vz at E26/27 and later stages. This temporally coincided with the thickening of the iz and was thought to represent neurogenesis of projection neurons through the canonical cascade (Winpenny et al., 2011). Given that the emergence of the svz has been proposed to associate with the ability to better control neurogenesis and to overcome the limitation of the vz in terms of neuronal output (ventricular choke hypothesis, Martínez-Cerdeño et al., 2016), we propose the existence of *Tbr2*-expressing intermediate progenitors in the OB of cats as of E26/27.

Interneurons settle in the feline OB around E24/25, as indicated by the upregulation of *Gad2* and *Er81* expression between the vz and the iz of the OB. In mice, most OB interneurons are generated after E12.5 (Díaz-Guerra et al., 2013) by dorsal striatal progenitors regulated by *Dlx2* and *Mash1* among others (Allen et al., 2007; Long et al., 2007). As mentioned above, we focused on *Er81*, which is crucial for the development of the dopaminergic interneurons (Cave et al., 2010), and *Gad2* that has been linked to periglomerular neurons (Esclapez et al., 1993, 1994). Our results further suggest that the OB may be capable of producing its own interneurons after E26/27; this conclusion is based on the high *Er81* expression detected within the vz, as in mice (Stenman et al., 2003a; Long et al., 2007).

While studying the genoarchitecture, we also defined the timing of several events and showed that staging equivalence should be studied concerning the process. Our results suggest a complex pattern of heterochronies between the mouse and the cat; represented by shifts in the timing of events (some events occurring earlier in the cat or vice versa). For instance, E22/23 is generally equivalent to the E11.5 stage of the mouse: olfactory bulb evagination, however, in the cat has already commenced at E22/23, while in mice this event takes place after E12 (Hinds, 1968; Imamura and Greer, 2013). On the other hand, the murine LP expresses *Nr4a2* as of E12.5 (Puelles, 2014), this upregulation in the feline LP is observed much later, after E28/29 (approximately a week later after the murine equivalent stage). Moreover, as expected, in the feline telencephalon developmental processes usually take much longer to complete; this may be used to accurately define distinct stages of a developmental process.

In summary, our results show that the overall genoarchitecture of the cat telencephalon is conserved, with territories topologically equivalent to those previously described for the mouse and other vertebrate species, following the prosomeric model and the derived ontology (Puelles et al., 2000, 2013, 2016a; Brox et al., 2004; Abellán and Medina, 2009; Abellán et al., 2014; Desfilis et al., 2018). Furthermore, they underline the heterochronous nature of developmental events in a comparative context between species. These results, along with the fact that the feline brain is gyrencephalic, suggest that cats can provide a useful animal model in the study of the brain in ontogenesis and evolution. To our knowledge, this is the first systematic analysis of the feline telencephalon genoarchitecture and we hope that it will provide a reference for future research, especially given the re-appearance of this species in neuroscience research (Hinova-Palova et al., 2019; Valdés-Cruz et al., 2019; Graff et al., 2020).

## Data Availability Statement

The original contributions presented in the study are included in the article/Supplementary Material, further inquiries can be directed to the corresponding author.

## Ethics Statement

Ethical review and approval was not required for the animal study because the feline embryos or fetuses were obtained from domestic cats referred to the Unit of Obstetrics and Surgery of the Companion Animal Clinic of the School of Veterinary Medicine, Faculty of Health Sciences, Aristotle University of Thessaloniki for preventive ovariohysterectomy. In case of pregnancy, the excised gravid uterus was not processed for incineration, but immediately incised - this process does not require approval.

## Author Contributions

NS conceptualized and designed the study, performed most of the experiments, and wrote the first draft of the manuscript. CV performed the ovariohysterectomies, the embryo dissection/initial staging, and reviewed the first draft. GS designed part of the experiments, supervised part of the work, and reviewed the manuscript. MG conceptualized and designed the study with NS, supervised the study, wrote the final manuscript, and provided project administration and funding acquisition.

## Conflict of Interest

The authors declare that the research was conducted in the absence of any commercial or financial relationships that could be construed as a potential conflict of interest.

## Publisher’s Note

All claims expressed in this article are solely those of the authors and do not necessarily represent those of their affiliated organizations, or those of the publisher, the editors and the reviewers. Any product that may be evaluated in this article, or claim that may be made by its manufacturer, is not guaranteed or endorsed by the publisher.

## Footnotes

1
https://www.ncbi.nlm.nih.gov/tools/primer-blast

2
https://developingmouse.brain-map.org/

3
https://gp3.mpg.de/

## Abbreviations

AA: Anterior Amygdala
AOB: Accessory olfactory bulb
AStr: Striatal amygdala
BAOT: Bed nucleus of the accessory olfactory tract
Ce: Central amygdalar nucleus
CGE: Caudal ganglionic eminence
Cl: Claustrum
CMS: Caudal migratory stream
cp: Cortical plate
CxH: Cortical hem
DB: Diagonal Band
Dg: Diagonal domain of the subpallium
DgSe: Septal subdivision of the Dg
DP: Dorsal Pallium
dS: Dorsal Striatal division
EA: Extended Amygdala
EPd: Dorsal Endopiriform Nucleus
GP: Globus Pallidus
Hi: Hippocampal anlage
hp1: Hypothalamic (or secondary prosencephalic) prosomere 1
hp2: Hypothalamic (or secondary prosencephalic) prosomere 2
ic: Internal capsule
ivf: Interventricular foramina
iz: Intermediate zone
LGE: Lateral Ganglionic Eminence
lot: Lateral olfactory tract
LP: Lateral Pallium
LPO: Lateral preoptic area
MCL: Mitral cell layer
MP: Medial Pallium
Acb: Nucleus Accumbens
NLOT: Nucleus of the lateral olfactory tract
OB: Olfactory bulb
ONL: Olfactory nerve layer
OT: Olfactory tubercule
Pal: Pallium
Pd: Pallidal domain
Pdm: Pallidal mantle
PdSe: Septal Pallidal subdivision
POA: Preoptic Area
POAm: Mantle of the POA
POASe: Septal subdivision of the POA
POH: Preoptic-hypothalamic border region
pp: Preplate
PSB: Pallial-subpallial boundary
PSe: Pallial Septum
PThE: Prethalamic Eminence
rf: Rhinal fissure
rld: Rostrolateral domain
SLEA: Sublenticular extended amygdala
SP: Subpallium
sp: Subplate
SPV: Supraoptic paraventricular region
Str: Striatal domain of the subpallium
StrSe: Septal striatal subdivision
svz: Subventricular zone
VAP: Ventropallial amygdalopiriform area
VAPvz: vz of the VAP
VAPm: Ventropallial amygdalopiriform area mantle
VP: Ventral Pallium
VPvz: vz of the VP
vS: Ventral Striatal division
vz: Ventricular zone.
