# Supplementary material for: Genoarchitectonic Compartmentalization of the Embryonic Telencephalon: Insights From the Domestic Cat
Source: Front Neuroanat. 2021 Dec 16;15:785541. doi: 10.3389/fnana.2021.785541 (PMC8716433; doi:10.3389/fnana.2021.785541)
Supplement: Supplementary file 1 [file Data_Sheet_1.pdf]

Supplementary Material

1 Supplementary Table 1

List of the primers that were used to amplify DNA fragments from the cat orthologue genes used in this study. Lowercase letters indicate non-feline sequences added to facilitate oriented cloning in the plasmid vectors, recognition sites of the restriction enzymes used for cloning (EcoRI: GAATTC, SalI: GACGTC, BamHI: GGATCC) are underlined.

**Gene (NCBI accession.version N.)**	**Forward primer**	**Reverse primer**
Mash1 (Ascl1) (XM_006933981.3)	catgaattcGTCACAAGTCAGCGTCCAA	catgtcgacTTGTGCGGTGACCCTATAAC
Dlx2 (XM_003990862.4)	cgggaatTCCAAGTTCAAGAAGATGTGGAAA	aatcgtcgaCAAAATGAGGTCATCCGCAAA
Emx1 (XM_023251768.1)	ttggaattCACTTCTACCCCTGGGT	aaggtcgacTTATTATTCCCATAGGGAAGG
Er81 (XM_019825535.2)	acagaatTCAGTGCCAGCACTATGTCA	acagtcgACGGTGCCTCTGTCTCACTT
Gad2 (XM_003988173.4)	gatgaattcGGCCAACTCTGTGACATGGA	cttgtcgacCGGACAGTGGCAATATGG
Lef1 (XM_011281702.3)	aaagaattCAGCGGAGCGGAGATTA	GAAAACCTGGACATGGAAGTG
Lhx2 (XM_011288451.3)	aatggatCCGCTGGGTCTTCCCTACTA	aatgtcgacTTCCTCGCTCAGTCCACAAA
Lhx6 (XM_023242685.1)	aaacgaattcGAGACACTGACCAACCCTGAGA	aatgtcgacAGGCTCAAGGCAGAGTGGGTA
Lhx7(8) (XM_023258831.1)	aacgaattcACAACTCTTGGACTCCAGCCCTT	gaggtcgacAAATTGCAGTTGCAAAATTCACAGA
Nr4a2 (Nurr1) (XM_006935294.4)	aatgaattCCTTGTGTTCAGGCGCAGTATGG	tatagtcgacAGGTGCGCACGCCATAGT
Pax6 (XM_023239672.1)	aatgaattcGTATCCGGGGACTTCTGTG	taagtcgacAGTGGTACAACACAGGACACAAT
Tbr1 (XM_023259391.1)	taagtcgacACTGGCTGCGACATGG	acatgaattcGTAGCCGCCTATGTCC
Tbr2 (Eomes) (XM_019810822.2)	taagtcgacATTGTCCCTGGAGGTCGGTA	gacggaaTTCAGTTACCTGCAGCAATCA

2 Supplementary Table 2

List of amplicons from the cat orthologue genes used in this study used for cloning on pBluescipt II KS (+). Digests with restriction enzymes and fill in reactions with DNA Polymerase I, Large (Klenow) Fragment were performed according to manufacturer's instructions (New England Biolabs).

**Gene**	**Amplicon modification**	**Vector pBluescipt II KS (+)**	**NCBI accession.version N. Amplicon position (5'-3')**
Mash1 (Ascl1)	EcoRI – SalI digest	EcoRI-SalI	XM_006933981.3 284-829
Dlx2	StuI – AfeI digest	EcoRV	XM_003990862.4 1136-1844
Emx1	AgeI – SalI digest	EcoRI-SalI	XM_023251768.1

	(AgeI sited filled in)	(EcoRI site filled-in)	7335-7777
Er81	SalI – EcoRI digest	EcoRI-SalI	XM_019825535.2<br>1878-3434
Gad2	EcoRI – HincII digest	EcoRI-HincII	XM_003988173.4<br>2051-3844
Lef1	EcoRI – SalI digest	EcoRI-SalI	XM_011281702.3<br>1159-1919
Lhx2	EcoRI – SmaI digest	EcoRI-EcoRV	XM_011288451.3<br>830-1423
Lhx6	EcoRI – SalI digest	EcoRI-SalI	XM_023242685.1<br>1789-3112
Lhx7/8	EcoRI – SalI digest	EcoRI-SalI	XM_023258831.1<br>1000-1542
Nr4a2 (Nurr1)	EcoRI – SalI digest	EcoRI-SalI	XM_006935294.4<br>542-1376
Pax6	EcoRI – AfeI digest	EcoRI-HincII	XM_023239672.1<br>636-1131
Tbr1	SalI – EcoRI digest	EcoRI-SalI	XM_023259391.1<br>1869-2696
Tbr2 (Eomes)	SalI – EcoRI digest	EcoRI-SalI	XM_019810822.2<br>1865-2718

3 Supplementary Table 3

List of events used to translate developmental time between murine and feline embryos, according to Workman et al., (2013).

**Event**	**Predicted value** (Rounded at 2 decimal places)	
	**Mouse**	**Cat**
Limbic system		
Post-proliferative zone appears in the medial pallium	11,37	22,85
Mitral cells - peak	12,05	24,48
Nucleus of lateral olfactory tract - peak	12,42	25,41
Olfactory tubercle generation-peak	13,41	27,85
Cortex		
Subplate - onset of neurogenesis	9,80	24,83
Neocortical Layer 1 emerges	12,63	25,92
Neurogenesis cortical layer VI - start (VC)	10,65	27,47
Cortical plate first observed/visible	10,77	27,85
Lhx6 first in cortex in GABAergic cells	13,41	27,85
External capsule appears	12,83	26,42
Internal capsule appears	13,36	27,74
Cortical subventricular zone (abventricular cells) - onset	11,66	30,63
Striatum		
Globus pallidus - peak	11,13	22,26
Caudoputamen – peak	12,15	24,73

4 Supplementary Figures

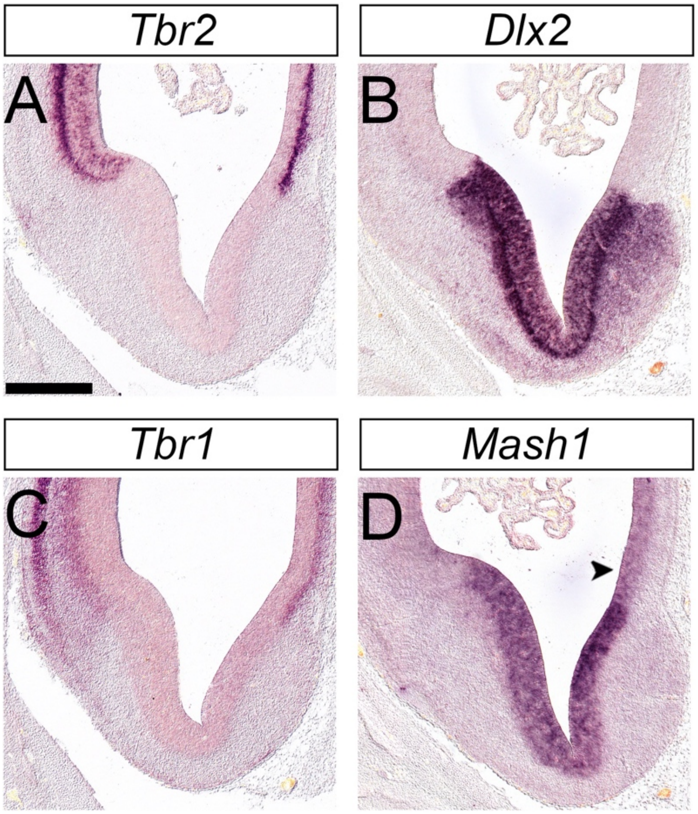

Supplementary Figure 1. *In situ* hybridization on coronal sections of rostral planes at E26/27 with *Tbr2* **(A)**, *Dlx2* **(B)**, *Tbr1* **(C)** and *Mash1* **(D)** probes. The subpallium expressed *Mash1* **(D)** in the vz and *Dlx2* **(B)** in both the vz and the svz, while the pallium expressed *Tbr2* **(A)** in the proliferative zones and *Tbr1* **(C)** in the mantle. Note the weak *Mash1* expression in the MP (arrowhead in **D**). Scale bar 500 µm.

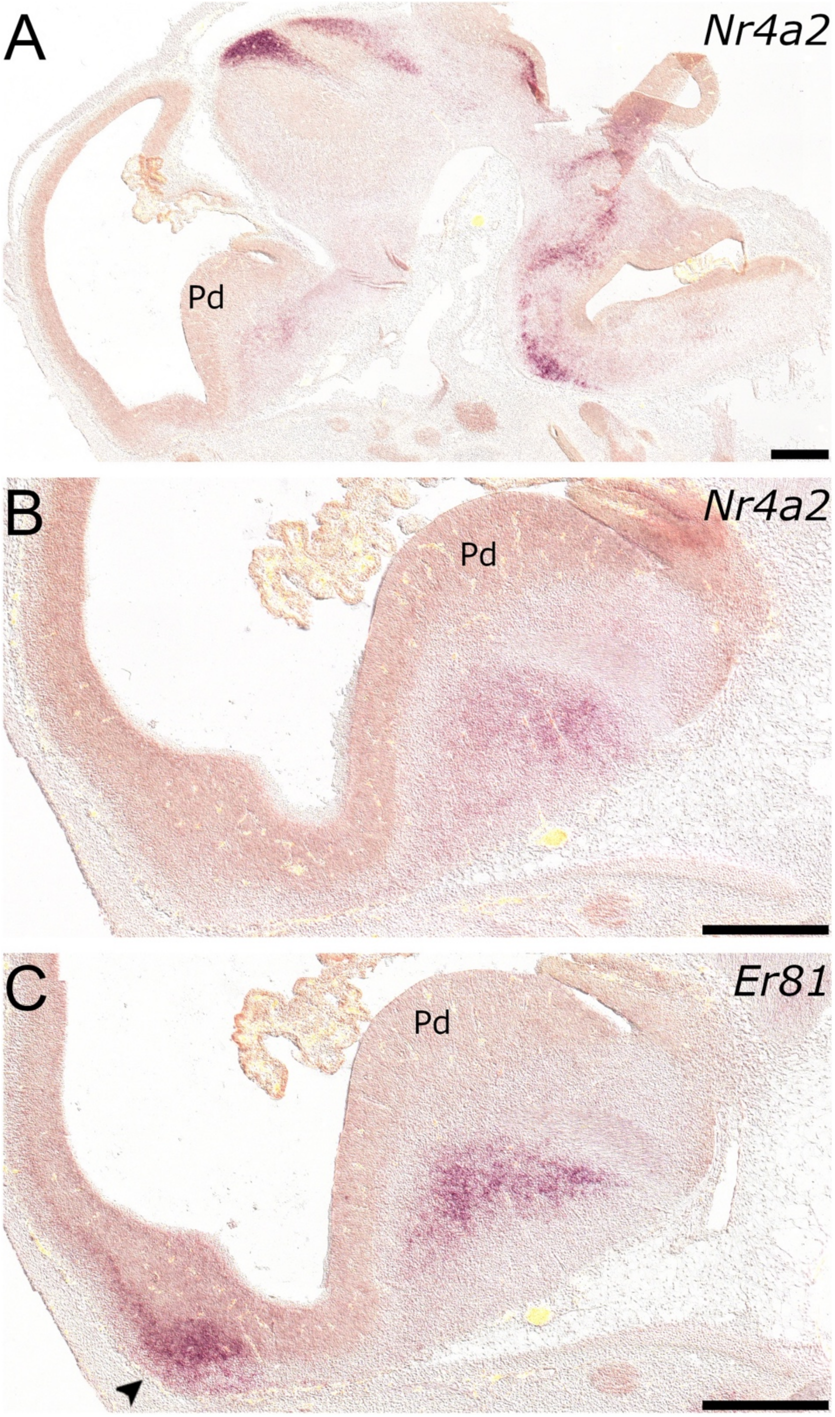

Supplementary Figure 2. *In situ* hybridization on sagittal sections of the CNS at E24/25 with the *Nr4a2* **(A-B)** or *Er81* **(C)** probes. *Nr4a2* was highly expressed in extratelencephalic areas **(A)**. In the telencephalon, it was expressed in the subpallial mantle **(B)**, along with *Er81* **(C)**. Arrowhead in **(C)** indicates the incipient OB. **(B)** and **(C)** are adjacent sections. Scale bar 500 µm.

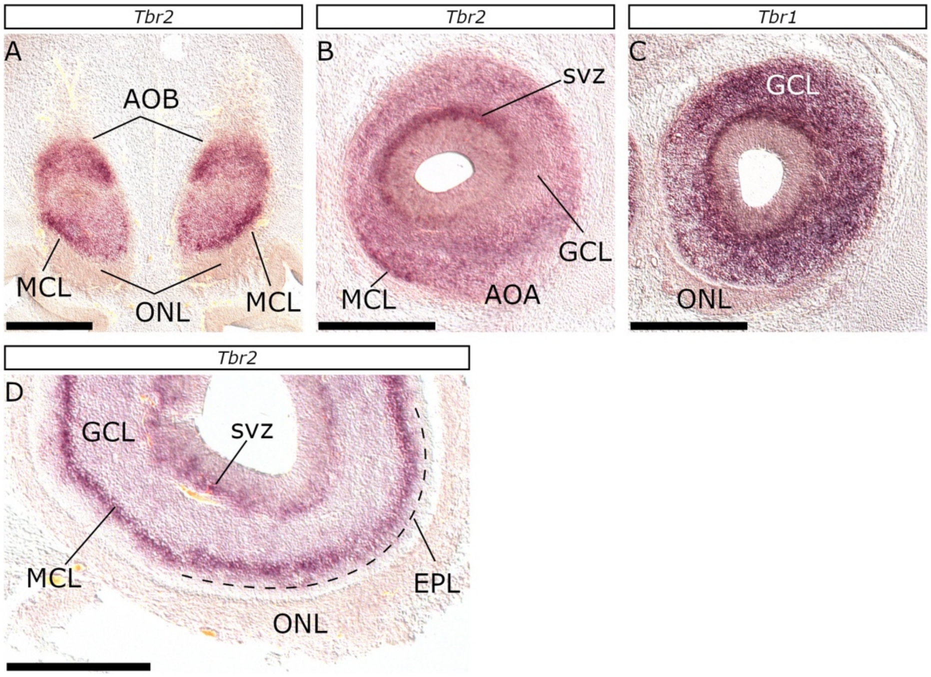

Supplementary Figure 3. *In situ* hybridization on coronal sections of the olfactory bulb at E26/27 **(A)**, E28/29 **(B,C)** and E34/35 **(D)** with the *Tbr2* **(A,B,D)** and *Tbr1* **(C)** probes. In **(A)** *Tbr2* demarcated the incipient MCL, in close contact with the ONL; *Tbr2* was also expressed dorsally, in the MCL of the AOB primordium. At E28/29 *Tbr2* **(B)** and *Tbr1* **(C)** were expressed in the svz (lining the basal aspect of the vz). At E34/35 **(D)**, *Tbr2* was expressed in the svz and the MCL, while the developing EPL was presented (dashed line) between the MCL and the ONL. Scale bars 500 μm.

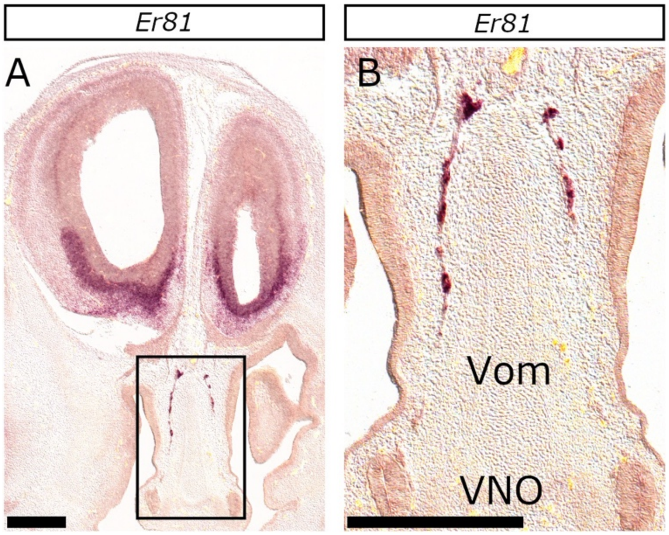

Supplementary Figure 4. *In situ* hybridization on coronal sections of the retrobulbar region with the *Er81* probe. **(B)** is close up of the boxed area in **(A)**. *Er81*-expressing cells migrating along the nervus terminals, represent GnRH-releasing neurons of the septum. Scale bar 500 μm.

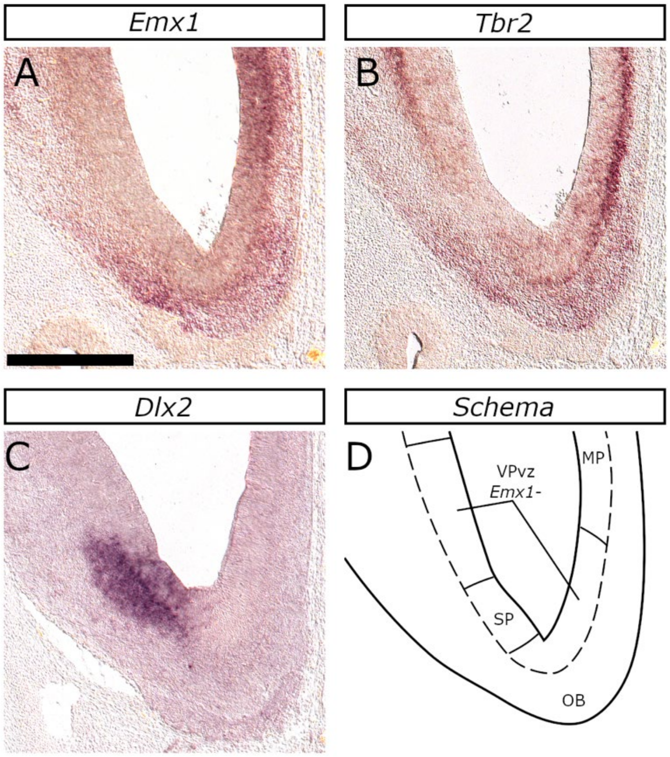

Supplementary Figure 5. *In situ* hybridization on coronal sections of the retrobulbar region at E26/27 with the *Emx1* **(A)**, *Tbr2* **(B)** and *Dlx2* **(C)** probes. **(D)** Schematic representation. The ventral-most septal region (ventral to the MP) is ventropallial featuring lack of *Emx1* expression. Scale bar: 500 µm.
